# HLA‐DR^+^ Schwann Cells Generate the Protumor Cancer‐Neuron‐Immune Niche in Head and Neck Squamous Cell Carcinoma

**DOI:** 10.1002/advs.76131

**Published:** 2026-06-15

**Authors:** Xiaoyan Meng, Zhonglong Liu, Shijian Zhang, Luoman Gan, Liren Cao, Jingjing Sun, Lingfang Zhang, Yue He

**Affiliations:** ^1^ Department of Oral Maxillofacial & Head and Neck Oncology College of Stomatology National Center for Stomatology National Clinical Research Center for Oral Diseases Shanghai Ninth People's Hospital Shanghai Jiao Tong University School of Medicine Shanghai Jiao Tong University Shanghai Key Laboratory of Stomatology Shanghai P. R. China; ^2^ Department of Oral and Maxillofacial Surgery Zhang Zhiyuan Academician Workstation Hainan Province Clinical Medical Center For Stomatology Hainan Western Central Hospital Shanghai Ninth People's Hospital Danzhou Hainan P. R. China; ^3^ Department of Oral and Maxillofacial Head and Neck Oncology Fengcheng Hospital of Shanghai Ninth People′s Hospital Group Shanghai P. R. China; ^4^ Department of Oral Pathology College of Stomatology National Center for Stomatology National Clinical Research Center for Oral Diseases Shanghai Ninth People's Hospital Shanghai Jiao Tong University School of Medicine Shanghai Jiao Tong University Shanghai Key Laboratory of Stomatology Shanghai P. R. China; ^5^ Suzhou Lingdian Biotechnology Co., Ltd Suzhou P. R. China

**Keywords:** head and neck squamous cell carcinoma, Il1β. macrophages, schwann cells, single‐cell RNA sequencing, Tregs, tumor microenvironment

## Abstract

Neural structures are widely distributed in the oral and maxillofacial region and play important roles in the progression of head and neck squamous cell carcinoma (HNSCC). Herein, we delineate the dynamic transition trajectory of Schwann cells during cancer initiation and progression and identify a Schwann cell subpopulation, HLA‐DR^+^ Schwann cell, that induced by cancer cells and enriched as HNSCC progression. With the in vitro coculture assay and a Schwann cell‐targeted gene engineering in vivo model, we demonstrate that Schwann cells are educated by HNSCC cells via the NRG1/ERBB3 axis, activate the downstream JAK/STAT signaling pathway, and acquire immunoregulatory and protumor phenotypes. Furthermore, HLA‐DR^+^ Schwann cells are found to secrete CCL2 to induce a protumor macrophage subpopulation (Il1β. Mph), which promotes CD4^+^ T cell accumulation via CXCL10/CXCR3 for HLA‐DR^+^ Schwann cells and assists them in shaping the cancer‐neuron‐immune niche and facilitating HNSCC progression. The tumor suppression effects of CCL2 inhibitor (Pirfenidone) and CXCR3 inhibitor (AMG487) are validated in the orthotopic tumor model. Our findings reveal the mechanism of how HLA‐DR^+^ Schwann cells generate cancer‐neuron‐immune niche, provide insights into tumor neurology, and lay foundations for therapeutics development for HNSCC patients.

## Graphical abstract

Hijacked and educated by HNSCC cells, HLA‐DR^+^ Schwann cells lost their normal neural‐related functions but acquired immunoregulatory phenotypes to promote CD4^+^ T cells transform into Tregs. HLA‐DR^+^ Schwann cells induced a macrophage subpopulation, Il1β. Mph, which functioned as the trap of CD4^+^ T cells for HLA‐DR^+^ Schwann cells and assisted them in shaping the cancer‐neuron‐immune niche and facilitating HNSCC progression.

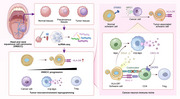

## 1 Introduction

Schwann cells are myelin cells of the peripheral nerves, and the main functions of these cells include neuroprotection, nutrition, and injury repair. Due to their high plasticity, Schwann cells play an important role in maintaining the homeostasis of the peripheral nervous system. The phenotype and function of Schwann cells are regulated by a variety of factors. For example, Neuregulin 1 (NRG1), which is a member of the epidermal growth factor family, is a key secretory factor that regulates the proliferation, differentiation, and phenotypic remodeling of Schwann cells by activating MAPK, PI3K, and JAK/STAT pathways [1, 2, 3].

With the development of tumor neurology, an increasing number of studies have focused on Schwann cells in the tumor microenvironment (TME) and their effects on tumor progression. For example, in solid tumors such as colon cancer, pancreatic cancer, lung cancer, and melanoma, tumor‐associated Schwann cells (TASs) reportedly increase tumor cell proliferation, induce epithelial‐mesenchymal transition, and promote tumor cell invasion and metastasis through the PI3K/AKT/GSK‐3β, Snail/Twist, and other tumor‐promoting signaling pathways [4, 5, 6, 7, 8]. In addition to directly interacting with tumor cells, Schwann cells can also interact with other cells in the TME. Sun et al. reported that in pancreatic cancer, TASs inhibit CD8^+^ T infiltration, thus leading to immunotherapy resistance [9]; Xue et al. proposed that TASs induce the phenotypic transition of cancer‐associated fibroblasts (CAFs) to a more malignant phenotype in pancreatic cancer [10]; Liu et al. elucidated that TASs impair CD8^+^ T cell function and promote immune checkpoint inhibitor therapy resistance in pancreatic ductal adenocarcinoma [11]. The abovementioned studies indicate that Schwann cells are important parts of the TME of neuron‐rich tumors, which can not only directly enhance the malignant biological behavior of tumor cells, but also facilitate tumor progression by interacting with other cells in the TME.

Due to the fact that the oral and maxillofacial regions are thoroughly distributed with peripheral sensory and motor nerves, such as the trigeminal nerve and tongue nerve, neural structures are also important components of the TME in head and neck squamous cell carcinoma (HNSCC) and may play crucial roles during tumorigenesis and progression. For example, neuropathy always occurs during the progression of HNSCC, which contributes to maxillofacial cancer pain; and the pathological manifestation of perineural invasion is a key factor that determines the clinical stage and prognosis of HNSCC patients [12]. A series of studies have demonstrated the tumor‐promoting role of neural components in HNSCC [13, 14, 15, 16, 17, 18]. Specifically, Gloria et al. reported that Schwann cells in HNSCC could facilitate tumor metastasis by secreting special extra cellular matrix molecules [17]. These studies highlight the importance of investigating neural structures to elucidate the mechanisms of HNSCC initiation, progression and therapy resistance.

In this study, we comprehensively investigated single‐cell RNA sequencing (scRNA‐seq) data of human HNSCC samples of different stages, including normal, precancerous, and tumor tissues. For the first time, we delineated the dynamic transition of Schwann cells during cancer initiation and progression. Notably, we identified a Schwann cell subpopulation, HLA‐DR^+^ Schwann cell, that induced by cancer cells and enriched as HNSCC progression. Hijacked and educated by HNSCC cells, HLA‐DR^+^ Schwann cells lost their normal neural‐related functions but acquired immunoregulatory phenotypes to promote CD4^+^ T cells transform into Tregs, which was validated via in vitro coculture assay, flow cytometry, and high‐throughput sequencing. Moreover, via a Schwann cell‐targeted gene engineering mouse model, an orthotopic tumor xenograft model, scRNA‐seq of mouse tumors, multiplex immunofluorescence (mIF) assay, in vitro and in vivo functional assays, we revealed that HLA‐DR^+^ Schwann cells induced a macrophage subpopulation, Il1β. Mph, which promotes CD4^+^ T cell accumulation for HLA‐DR^+^ Schwann cells and assisted them in shaping the cancer‐neuron‐immune niche and facilitating HNSCC progression. Our findings revealed the mechanism of how HLA‐DR^+^ Schwann cells reprogrammed the TME of HNSCC, provided insights into tumor neurology, and laid the foundations for therapeutics development for HNSCC patients.

## 2 Results

### 2.1 Identification of HLA‐DR^+^ Schwann Cells with Rewired Phenotypes in the TME of HNSCC

**FIGURE 1:**
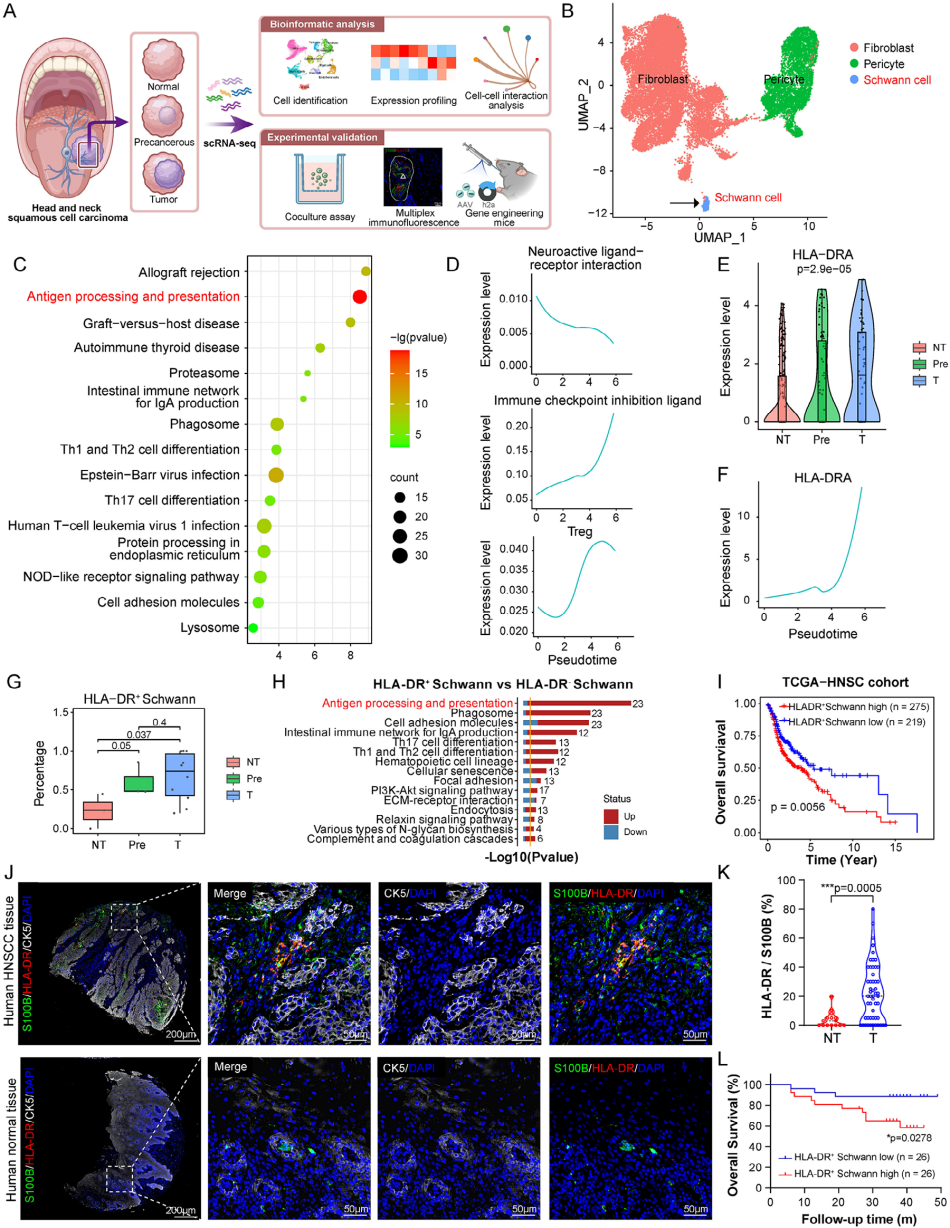
Identification of HLA‐DR^+^ Schwann cells with rewired phenotypes in the TME of HNSCC. (A) Illustration of the workflow of this study. (B) UMAP plot of stromal cells from 19 samples, showing 3 cell types (fibroblast, pericyte, and Schwann cell). (C) Dot plots of the top 15 pathways enriched in Schwann cells in the T stage. (D) Expression levels of different scores of Schwann cells along the pseudotime axis. (E) Violin plots showing the expression level of *HLA‐DRA* in Schwann cells in different stages. (F) Expression level of *HLA‐DRA* of Schwann cells along the pseudotime axis. (G) Box plots showing HLA‐DR^+^ Schwann cell percentage in total Schwann cells in different stages. (H) KEGG pathway analysis results of DEGs of HLA‐DR^+^ Schwann cells compared to HLA‐DR^−^ Schwann cells. (I) The Kaplan‐Meier OS curves of samples with high (n = 275) and low (n = 219) HLA‐DR^+^ Schwann cell infiltration in the TCGA‐HNSC cohort (n = 494). (J‐K) Representative images of mIF staining of HLA‐DR^+^ Schwann cells in human HNSCC tumor and normal tissues. Scale bar, 200 µm, 50 µm. Green: S100B, red: HLA‐DR, grey: CK5, blue: Dapi. The quantitative results are shown in (K) (n = 67). (L) The Kaplan‐Meier OS curves of samples with high (n = 26) and low (n = 26) HLA‐DR^+^ Schwann cell infiltration in the validation cohort (n = 52), related to (J‐K). *P* values were calculated by the hypergeometric test in (C,H), by one‐way ANOVA in (E), by one‐side Wilcoxon test in (G), by two‐sided Student's *t*‐test in K, and by two‐sided log‐rank test in (I,L). ^*^ *p <* 0.05, ^***^ *p* < 0.001.

To characterize the diverse TME of HNSCC, we conducted scRNA‐seq of samples from 12 HNSCC patients, including normal tissues (NT), precancerous tissues (Pre), and tumor tissues (T), as we have previously reported (Figure 1A) [19]. Among stromal cells, we identified fibroblasts (n = 15, 276), which were defined by *DCN* and *COL1A1*; pericytes (n = 5, 307), which were defined by *MCAM* and *TAGLN*; and Schwann cells (n = 234), which were defined by *CDH19* and *NGFR* (Figure 1B). Given the high prevalence of neural components in the head and neck region, here Schwann cells caught our attention, which were also identified in other HNSCC scRNA‐seq datasets (Figure S1A, GSE164241 [20] and GSE188737 [21]). Interestingly, though Schwann cell proportion seemed to be comparable among three groups (Figure S1B), Schwann cells in T group exhibited different transcriptomic features compared to their counterparts in NT and Pre: expression levels of neuron‐related genes were downregulated, such as “Neuroactive ligand‐receptor interaction”, and expression levels of immunoregulatory genes were upregulated, such as “Antigen processing and presentation”, “Immune checkpoint inhibition ligand”, and “Treg”. We performed pseudotime analysis of Schwann cells and found consistent results that, as HNSCC initiation and progression, Schwann cells underwent phenotype rewiring, losing neural regulating functions and acquiring immune regulating phenotypes (Figure 1C,D; Figure S1C,D). Major histocompatibility complex class II molecules (MHC II) are responsible to antigen processing and presentation. We checked the expression levels of HLA family genes in Schwann cells in different stages and found that they were upregulated in tumors, especially genes belonging to MHC II subclass (Figure S1E,F). Specifically, the expression level of *HLA‐DRA* in Schwann cells was found to gradually increase from NT group to pre group, and further to T group, and thus was selected as a marker for dysfunctional Schwann cells in HNSCC tissues (Figure 1E,F). Based on the expression level of *HLA‐DR*, Schwann cells were clustered into HLA‐DR^−^ and HLA‐DR^+^ subpopulations, and we focused on HLA‐DR^+^ Schwann cells for further investigation (Figure S1G). Schwann cells could be divided into myelinating and non‐myelinating subclusters according to the classic classification [9], and HLA‐DR^+^ Schwann cells belong to the nonmyelinating subcluster (Figure S1H), which again indicates the dysfunctional status of HLA‐DR^+^ Schwann cells in HNSCC. As shown in Figure 1G,H, compared to HLA‐DR^−^ Schwann cells, the percentage of HLA‐DR^+^ Schwann cell increased with HNSCC progression and upregulated genes related to antigen processing and presentation, which was consistent with our previous analysis (Figure 1C,D; Figure S1C,D). Notably, HLA‐DR^+^ Schwann cell was associated with worse overall survival (OS) in HNSCC (*p* = 0.031, Figure 1I; Figure S1I). The clinical prognostic relevance of HLA‐DR^+^ Schwann cells highlighted the significance of investigating their rewired phenotype and revealing the underlying mechanism.

Then, we turned to the in‐house tissue microarray (TMA) cohort for validation. S100B was selected as the marker for Schwann cells [17], and CK5 was selected as the marker for epithelial cells. We characterized the presence of HLA‐DR^+^ Schwann cells in HNSCC tumor tissues (T) and normal tissues (NT) via mIF assay. HLA‐DR^+^ Schwann cells could be found in HNSCC tumor tissues, close to malignant epithelial cells. In contrast, although Schwann cells were located among normal epithelial cells in the normal tissues, they were almost negative in HLA‐DR expression. As for the quantitative results, the ratio of HLA‐DR^+^ Schwann cells was significantly higher in T samples (*p* = 0.0005, Figure 1J,K). Moreover, when divided with the HLA‐DR^+^ Schwann cell ratio, patients with higher HLA‐DR^+^ Schwann cell ratios were associated with more advanced disease stages (*p* = 0.0245, Figure S2A), poorer OS (*p* = 0.0278, Figure 1L), and poorer metastasis‐free survival (MFS) (*p* = 0.0045, Figure S2B), which suggested a clinical correlation between HLA‐DR^+^ Schwann cells and unfavorable outcomes in HNSCC patients. To simulate the original HNSCC initiation and progression process in vivo, we also constructed an autochthonous HNSCC mouse model as previously described [22]. We obtained various tissue samples, including NT, pre, and T tissues. In the mouse validation cohort, the HLA‐DR^+^ Schwann cell ratio (IA/IE in mice corresponded to HLA‐DR in human) increased from the NT, then to the pre, and finally to the T samples, which was consistent with the results in the human HNSCC cohort and further indicated that Schwann cells were associated with HNSCC initiation and progression (Figure S2C,D). In addition, due to the fact that Schwann cells are widely distributed in normal tissues such as intestinal, lung, and kidney [23], we evaluated human normal tissues via mIF assay and observed that although Schwann cells were indeed present in them, they did not express HLA‐DR genes (Figure S2E–G), which suggested that the immunoregulatory phenotype of Schwann cells is the specific result of tumor educating. In summary, we identified HLA‐DR^+^ Schwann cells in both human and mouse HNSCC tissues with rewired phenotypes, which were associated with poor clinical outcomes in HNSCC patients.

### 2.2 HNSCC Cells Educate Schwann Cells via NRG1/ERBB3

**FIGURE 2:**
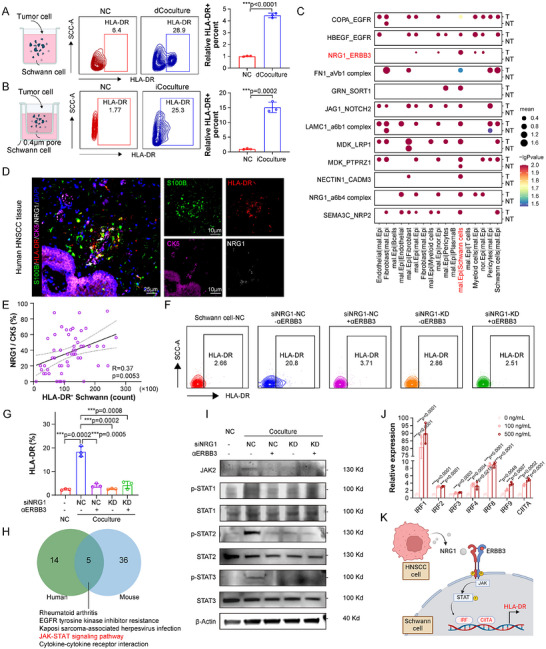
HNSCC cells educate Schwann cells via NRG1/ERBB3. (A,B) Schematic illustration (left), representative flow cytometry images (middle), and quantitative results of the HLA‐DR^+^ cell ratio (right) of human Schwann cells that were (A) directly cocultured and (B) indirectly cocultured with HNSCC cells (n = 3) (Schematic illustrations were created with BioRender.com). (C) Dot plots showing the cell–cell interaction strengths of pairs between the main cell types and Schwann cells. (D,E) Representative images of mIF staining of HLA‐DR^+^ Schwann cells and NRG1^+^ malignant epithelial cells in human HNSCC tumor samples. Scale bar, 25 µm, 10 µm. Green: S100B, red: HLA‐DR, purple: CK5, grey: NRG1, blue: Dapi. HLA‐DR^+^ Schwann cells were indicated by asterisks, and NRG1^+^ malignant epithelial cells were indicated by arrows. The Pearson correlation results are shown in (E) (n = 55). (F,G) Representative flow cytometry images (F) and quantitative results (G) of the HLA‐DR^+^ cell ratio of Schwann cells subjected to different treatments (n = 3). (H) Venn diagrams showing enriched pathways of DEGs of HLA‐DR^+^ compared to HLA‐DR^−^ Schwann cells in human (left) and mouse (right) cells. The intersection pathway results are listed below. (I) WB analysis of the levels of JAK‐STAT pathway proteins in Schwann cells with different treatments. (J) MRNA expression levels of the indicated genes in Schwann cells treated with rhNRG1 in different concentrations (n = 3). (K) Schematic illustration of how HNSCC tumor cells educate Schwann cells (created with BioRender.com). *P* values were calculated by empirical shuffling in C, and by two‐sided Student's *t*‐test in (A,B,G,J). ^*^ *p <* 0.05, ^**^ *p* < 0.01, ^***^ *p* < 0.001.

Subsequently, we investigated how Schwann cells acquired specific immunoregulatory phenotypes in HNSCC tumor tissues. Given that malignant epithelial cells are the primary components of tumor tissues and HLA‐DR^+^ Schwann cells were found to be close to the malignant cells in the mIF assay (Figure 1J), we first focused on tumor‐Schwann cell interactions. Direct (dCoculture) and indirect (iCoculture) coculture models were established with a Transwell device: for direct coculture, human HNSCC cells were added to the well and contacted the Schwann cells directly; for indirect coculture, human HNSCC cells were seeded in a Transwell device with 0.4 µm pores and the device was then placed into the well with Schwann cells; Schwann cells not cocultured with HNSCC cells were set as control (NC) (Figure 2A,B). Via flow cytometry assay, we observed that both direct and indirect coculture with human HNSCC cells induced HLA‐DR expression in human Schwann cells (dCoculture: *p* < 0.0001, iCoculture: *p* = 0.0002, Figure 2A,B; Figure S3A,B). The results of the mouse Schwann cell assay were consistent with those of the human Schwann cell assay (Figure S3C,D). These results suggested that malignant cells may educate Schwann cells via secretory factors rather than via direct cell–cell contact pairs.

We next screened tumor‐derived secretory factors via the CellChat algorithm. Among ligand‐receptor pairs that exhibited stronger strengths in tumor tissues than in normal tissues, NRG1 and ERBB3 were relatively exclusively expressed in malignant epithelial cell and Schwann cell. Therefore, NRG1/ERBB3 was selected for further validation (Figure 2C; Figure S4A,B). In the validation cohort, NRG1^+^ tumor cells and HLA‐DR^+^ Schwann cells could be observed to colocalize with each other in HNSCC tumor tissues, and their infiltration ratio exhibited positive correlation with each other (R = 0.37, *p* = 0.0053, Figure 2D,E). Then, the tumor‐Schwann cell coculture model was used to validate cell–cell interaction in vitro. As shown in Figure 2F,G, HLA‐DR^+^ cell ratio of Schwann cells increased after cocultured with HNSCC cells (*p* = 0.0002). However, when *NRG1* was knockdown in HNSCC cells with small interfering RNA (siRNA) or ERBB3 blockade antibody (αERBB3) was added in the cell medium, HLA‐DR^+^ cell ratio significantly decreased to the level of control Schwann cell, which suggested that malignant epithelial cells induce *HLA‐DR* expression of Schwann cells via NRG1/ERBB3.

Moreover, to explore the molecular mechanism involved in this educating process, we performed bulk RNA‐seq on human and mouse Schwann cells. Gene Ontology (GO) and Kyoto Encyclopedia of Genes and Genomes (KEGG) pathway enrichment analyzes were conducted on the differentially expressed genes (DEGs) of Schwann cells after cocultured with HNSCC cells and we identified five conserved pathways by intersecting upregulated pathways in human and mouse Schwann cells, among which the “JAK‐STAT signaling pathway”, as was widely reported to be associated with MHC II complex synthesis, attracted our attention (Figure 2H; Figure S5A–D) [24]. Again, we used the coculture model and evaluated the activation of the JAK‐STAT signaling pathway via Western Blotting (WB). In Schwann cells cocultured with tumor cells, we observed the upregulation of the JAK2 protein and increased phosphorylation of STAT2 and STAT3, while *NRG1* knockdown in HNSCC cells and αERBB3 administration led to a decrease of them (Figure 2I). The *IRF* genes family and the transcription factor *CIITA* are downstream genes of the JAK‐STAT signaling pathway and can induce the expression of MHC II genes [25]. After treated with recombinant human NRG1 (rhNRG1) in different concentrations, we observed significant induction of *IRF* gene family expression and upregulation of *CIITA* in human Schwann cells (Figure 2J). Meanwhile, the treatment of JAK‐STAT signaling inhibitor [26] significantly reduced the proportion of HLA‐DR^+^ Schwann cell (Figure S5E,F). The above results suggested that HNSCC cells secrete NRG1, combining with ERBB3 on Schwann cells, which activates the JAK‐STAT signaling pathway (STAT2 and STAT3) and induces phenotype transition of Schwann cells (Figure 2K).

### 2.3 HLA‐DR^+^ Schwann Cells Facilitate HNSCC Progression

**FIGURE 3:**
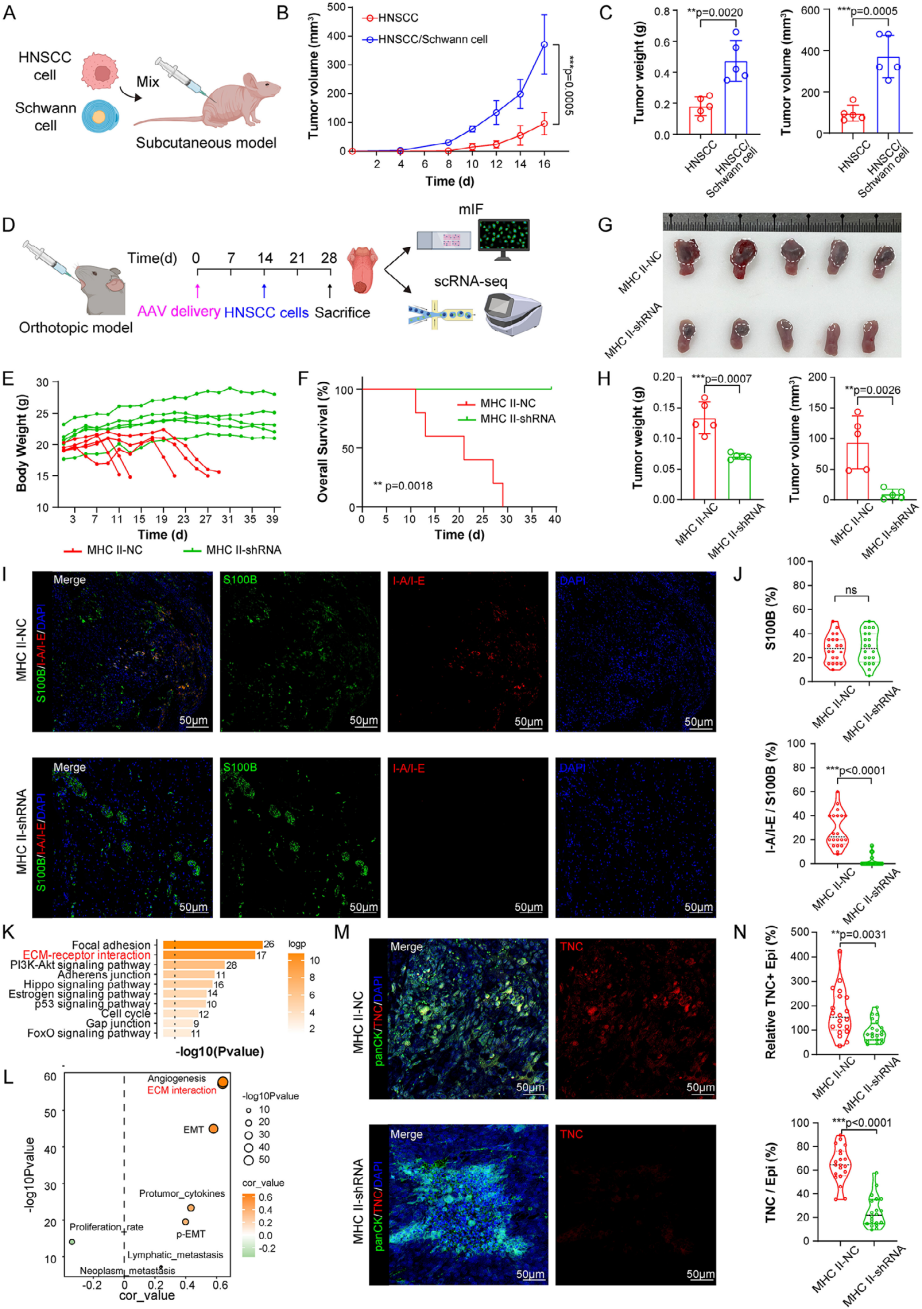
HLA‐DR^+^ Schwann cells facilitate HNSCC progression. (A) Schematic diagram of the subcutaneous xenograft tumor model (created with BioRender.com). (B,C) Tumor growth curves (B), tumor weights (C, left), and tumor volumes (C, right) of C3H/HeJ mice injected with mouse HNSCC cells with or without Schwann cells (n = 5). (D) Schematic diagram of the orthotopic xenograft tumor model and the Schwann cell‐targeted MHC II knockdown model (created with BioRender.com). (E,F) Body weight curves (E) and survival curves (F) of the MHC II‐NC and MHC II‐shRNA groups (n = 5). (G,H) Gross images (G), tumor weights (H, left), and tumor volumes (H, right) of the mice sacrificed 14 days after tumor cell injection (n = 5). (I) Representative images of mIF staining of HLA‐DR^+^ Schwann cells in tumors from the MHC II‐NC and MHC II‐shRNA groups. Scale bar, 50 µm. Green: S100B, red: I‐A/I‐E, blue: Dapi. (J) Quantitative results of Schwann cell ratio (upper) and HLA‐DR^+^ Schwann cell ratio (lower) in tumors from the MHC II‐NC and MHC II‐shRNA groups (n = 20). (K) KEGG pathway analysis of the predicted target genes expressed in cancer cells. (L) Bubble plots showing correlation of cancer biological activity signatures and HLA‐DR^+^ Schwann cell signature. (M) Representative images of mIF staining of TNC^+^ epithelial cells in tumors from the MHC II‐NC and MHC II‐shRNA groups. Scale bar, 50 µm. Green: panCK, red: TNC, blue: Dapi. (N) Quantitative results of TNC^+^ epi counts (upper) and ratio (lower) in tumors from the MHC II‐NC and MHC II‐shRNA groups (n = 20). *P* values were calculated by two‐sided Student's *t*‐test in (B,C,H,J,N), by two‐sided log‐rank test in (F), and by hypergeometric test in (K,L). ^**^ *p* < 0.01, ^***^ *p* < 0.001.

We have validated the presence of HLA‐DR^+^ Schwann cells in HNSCC with acquired immunoregulatory functions, which were consequences of cancer cell educating. To investigate the role of HLA‐DR^+^ Schwann cells in HNSCC progression, we performed the following in vitro and in vivo experiments. First, we used the indirect cell coculture assay to evaluate cancer cell viability with or without Schwann cells, and we found that HLA‐DR^+^ Schwann cells did not enhance the proliferation capability of HNSCC cells (Figure S6A). Then, we used a subcutaneous tumor xenograft model: mouse HNSCC cell line SCC7 cells were subcutaneously injected with or without mouse Schwann cells (at a ratio of 20:1 as previously reported [10]). Both immunodeficient (Balb/c nude) and immunocompetent (C3H/HeJ) mice were evaluated for tumor volume and tumor weight to involve the immune microenvironment into the investigation. Interestingly, in immunodeficient mice, the mixture of tumor cells and Schwann cells did not accelerate tumor progression, whereas in immunocompetent mice, mixture injection‐derived tumors grew faster than their counterparts (Figure 3A–C; Figure S6B). These results indicated that the tumor‐promoting effects of HLA‐DR^+^ Schwann cells depended on the immune microenvironment of HNSCC, which was consistent with our previous in silico results that HLA‐DR^+^ Schwann cells exhibited immunoregulatory characteristics.

Next, to better simulate the development of HNSCC in the oral environment, we used the orthotopic tumor xenograft model and knockdown *h2a* gene (corresponding to *HLA‐DR* in human) via the delivery of an adeno‐associated virus (AAV)‐expressing. Schwann cell‐targeted short hairpin RNA (shRNA) targeting the mouse *h2a* gene (encodes the classical MHC II molecule, referred as MHC II‐shRNA, the control group was referred as MHC II‐NC) via orthotopic delivery (Figure 3D) [27]. Orthotopic delivery of the AAV showed good targeting capability and little systematic toxicity, as weak fluorescent signals were detected in other major organs, and the hematoxylin and eosin (H&E) staining of major organs from MHC II‐shRNA group exhibited normal histological morphology as their counterparts (Figure S6C,D). Notably, mice injected with HNSCC cells on their tongues lost weight gradually, while mice depleted HLA‐DR^+^ Schwann cells exhibited stable body weight curves and prolonged survival times (*p* = 0.0018) (Figure 3E,F). After 2 weeks of tumor transplantation, tongues of mice of MHC II‐NC and MHC II‐shRNA groups were collected for further evaluation. We found that HLA‐DR^+^ Schwann cell depletion significantly decreased HNSCC tumor weight (*p =* 0.0007) and volume (*p* = 0.0026) in vivo (Figure 3G,H; Figure S6E). Via mIF assay, we validated that the knockdown of *h2a* expression (I‐A/I‐E) in Schwann cells did not affect the total Schwann cell ratio (S100B: ns) but effectively decreased the HLA‐DR^+^ Schwann cell ratio (I‐A/I‐E/S100B: *p* < 0.0001) in the TME of HNSCC (Figure 3I,J). In the meantime, the gene‐editing strategy showed good cell‐type specificity on Schwann cells and had few effects on professional antigen presenting cells (APCs) or fibroblasts (Figure S6F–H). These results confirmed the effectiveness of the mice model. Given that HLA‐DR^+^ Schwann cells might not directly promote cancer cell proliferation as previously shown (Figure S6A), we would like to investigate how HLA‐DR^+^ Schwann cell affects other biological activities of cancer cells. Via interaction analysis using Nichnet algorithm and correlation analysis of HLA‐DR^+^ Schwann cell signature and tumor biological activity signatures, we found “ECM receptor interaction” pathway was enriched in both results (Figure 3K,L). *Tenascin C (TNC)* was among the top genes and was selected as the marker for this pathway. Via mIF assay, we validated that in MHC II‐NC group, cancer cells represented higher TNC expression level than cancer cells in MHC II‐shRNA group, which suggested that HLA‐DR^+^ Schwann cells might promote ECM interaction of cancer cells with stromal components (Figure 3M,N). Collectively, these results validated pro‐tumor effects of HLA‐DR^+^ Schwann cells in HNSCC.

### 2.4 HLA‐DR^+^ Schwann Cells Drive CD4^+^ T Cells into Tregs in HNSCC

**FIGURE 4:**
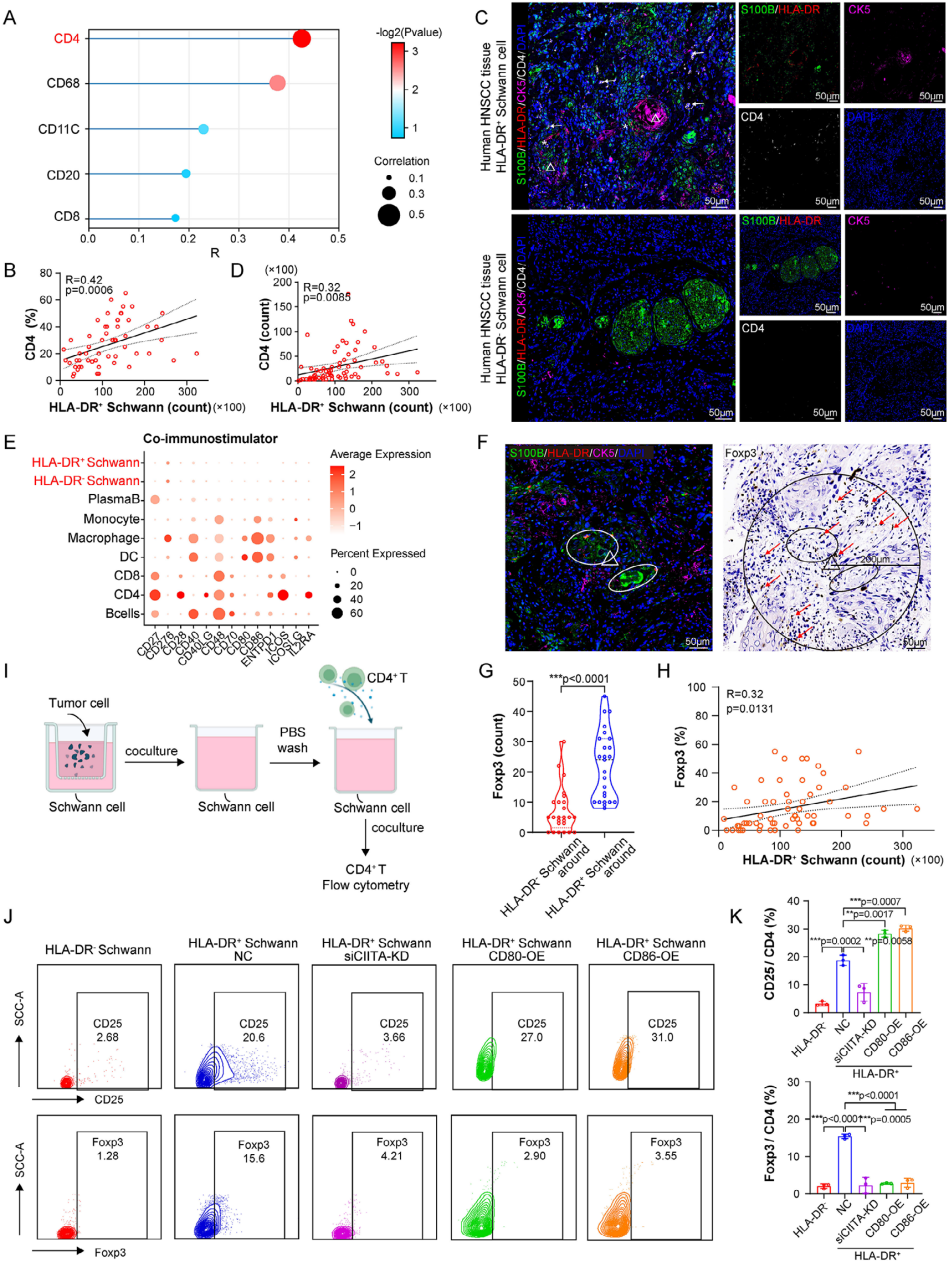
HLA‐DR^+^ Schwann cells drive CD4^+^ T cells into Tregs in HNSCC. (A) Lollipop plots showing the correlation of HLA‐DR^+^ Schwann cell count and ratio of different immune cell types in the validation cohort. (B) Pearson correlation results of the HLA‐DR^+^ Schwann cell count and CD4^+^ T cells in the validation cohort (n = 61). (C,D) Representative images of mIF staining of HLA‐DR^+^ Schwann cells and CD4^+^ T cells in human HNSCC tumor samples. Scale bar, 50 µm. Green: S100B, red: HLA‐DR, purple: CK5, grey: CD4, blue: Dapi. HLA‐DR^+^ Schwann cells were indicated by asterisks, epithelial cells were indicated by triangles, and CD4^+^ T cells were indicated by arrows. The Pearson correlation results are shown in (D) (n = 68). (E) Dot plots showing the expression levels of co‐immunostimulator genes in different cell types in the in‐house scRNA‐seq cohort. (F) Representative images of mIF staining of HLA‐DR^+^ Schwann cells (left) and IHC staining of Tregs (right) in human HNSCC tumor samples. Scale bar, 50 µm. Green: S100B, red: HLA‐DR, purple: CK5, blue: Dapi. (G) Violin plots showing Treg counts in the HLA‐DR^−^ and HLA‐DR^+^ Schwann cell around regions (n = 25). (H) The Pearson correlation results of HLA‐DR^+^ Schwann cell count and Treg ratio in the validation cohort (n = 61). (I) Schematic illustration of Schwann cell‐CD4^+^ T cell coculture model (created with BioRender.com). (J,K) Representative flow cytometry images (J) and quantitative results (K) of CD25^+^ cell (upper) and Foxp3^+^ cell (lower) ratio of CD4^+^ T cells in different groups (n = 3). *P* values were calculated by two‐sided Student's *t*‐test in (G,K). ^**^ *p <* 0.01, ^***^ *p* < 0.001.

Since the results of the above in vivo model suggested that the protumor effects of HLA‐DR^+^ Schwann cells relied on the immune microenvironment of HNSCC (Figure 3), we aimed to investigate how HLA‐DR^+^ Schwann cells remodel the immune cells. First, we investigated the correlations of HLA‐DR^+^ Schwann cells and different immune cells in the in‐house human TMA cohort. Immunohistochemistry (IHC) staining of CD4^+^ T cells (CD4), CD8^+^ T cells (CD8), macrophages (CD68), dentritic cells (DCs, CD11c), and B cells (CD20) was performed, and the correlations between immune infiltration ratios and HLA‐DR^+^ Schwann counts were subsequently calculated. Among five immune cells, CD4^+^ T cell showed the highest correlation with HLA‐DR^+^ Schwann cells (R = 0.42, *p* = 0.0006, Figure 4A,B; Figure S7A–C). Moreover, we delineated the TME via mIF assay and validated the IHC results: Schwann cells that were close to tumor epithelial cells (triangle marked) upregulated HLA‐DR (HLA‐DR^+^ Schwann cell, asterisk marked), and in niches with high HLA‐DR^+^ Schwann cell ratio, increased CD4^+^ T cell infiltration (arrow marked) was also observed (R = 0.32, *p* = 0.0085, Figure 4C,D).

According to previous reports, the full activation of CD4^+^ T cells requires two stimulating signals: the MHC II complex presenting antigens as the first signal and the costimulatory molecules as the second signal, which could lead to further clonal expansion of T cells [28]. However, unlike other professional APC (pAPC) (e.g., macrophages, DCs), HLA‐DR^+^ Schwann cells lacked classical costimulatory molecules (e.g., *CD80*, *CD86*, and *CD40*), which can lead to T cell anergy or the induction of Tregs [29] (Figure 3E). In the TCGA‐HNSC cohort, patients with higher HLA‐DR^+^ Schwann cell infiltration were found with higher Treg infiltration levels (R = 0.3443, *p* < 0.0001, Figure S7D) and higher immune suppression scores (Figure S7E). Based on the above results, we then investigated the correlation of HLA‐DR^+^ Schwann cells and Tregs from the level of spatial localization via mIF and IHC staining: We identified HLA‐DR^+^ Schwann cells in tumor samples and annotated regions within a radius of 200 µm centered with HLA‐DR^+^ Schwann cells as “HLA‐DR^+^ Schwann cell around region” and regions within a radius of 200 µm centered with HLA‐DR^−^ Schwann cells as “HLA‐DR^−^ Schwann around region”. By comparing HLA‐DR^+^ Schwann cell around with HLA‐DR^−^ Schwann cell around regions, we confirmed that Tregs tend to infiltrate in the HLA‐DR^+^ Schwann cell around region (*p* < 0.0001, Figure 4F,G) and that the Treg ratio was positively correlated with the number of HLA‐DR^+^ Schwann cells (R = 0.32, *p* = 0.0131, Figure 4H). Finally, we investigated the effects of HLA‐DR^+^ Schwann cells on CD4^+^ T cell transformation using an in vitro coculture model: Schwann cells were cocultured with tumor cells first to generate HLA‐DR^+^ Schwann cells as previously described, then CD4^+^ T cells were cocultured with HLA‐DR^+^ Schwann cells (HLA‐DR^−^ Schwann cells as control), and their immune phenotypes were further evaluated with flow cytometry assay (Figure 4I). Since CIITA is a major regulator for MHC‐II expression and CD80 and CD86 are two key costimulatory molecules, we also generated *CIITA* knockdown and *CD80, CD86* overexpression (OE) in HLA‐DR^+^ Schwann cell to evaluate whether antigen‐presenting signals is crucial for inducing Tregs [30]. After cocultured with HLA‐DR^+^ Schwann cells for 48 h, a higher expression ratio of early activation marker, CD25, was found in CD4^+^ T cells (*p* = 0.0002), which suggested the antigen presenting effects of HLA‐DR^+^ Schwann cells. In the meantime, more CD4^+^ T cells transferred into Tregs (*p* < 0.0001). Interestingly, knockdown of *CIITA* in HLA‐DR^+^ Schwann cells could induce a suppression of both CD4^+^ T cell activation and Treg transformation (CD25^+^: *p =* 0.0058, Foxp3^+^: *p* = 0.0005) while overexpression of *CD80* and *CD86* reverses the Treg transformation trend (CD80 and CD86: *p* < 0.0001) (Figure 4J,K). Taken together, these results suggested that HLA‐DR^+^ Schwann cells might be a special immune‐regulating cell that can induce Treg formation in HNSCC.

### 2.5 HLA‐DR^+^ Schwann Cells Induce the Protumor Macrophage Subtype Il1β. Mph via CCL2

**FIGURE 5:**
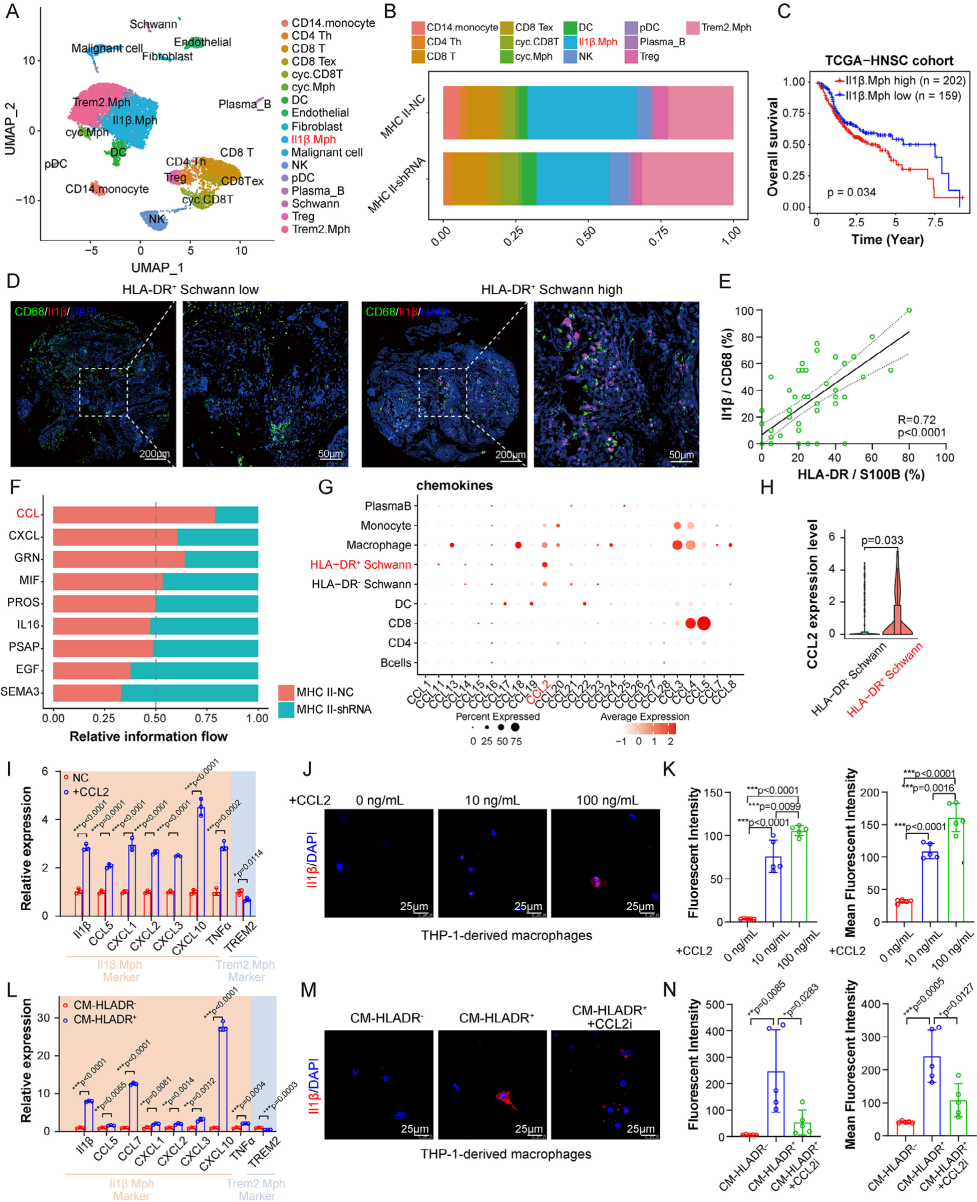
HLA‐DR^+^ Schwann cells induce the protumor macrophage subtype Il1β. Mph via CCL2. (A) UMAP plots of cells from tumors of MHC II‐NC and MHC II‐shRNA mice. (B) Bar plots showing different cell ratios between the groups. (C) The Kaplan–Meier OS curves of samples with high (n = 202) and low (n = 159) Il1β. Mph infiltration in the TCGA‐HNSC cohort (n = 361). (D) Representative images of mIF staining of Il1β. Mph in human HNSCC tumor samples with high and low HLA‐DR^+^ Schwann cell infiltration. Scale bar, 50 µm, 20 µm. Green: CD68, red: Il1β, blue: Dapi. (E) The Pearson correlation results of HLA‐DR^+^ Schwann cell ratio and Il1β. Mph ratio in the validation cohort (n = 51). (F) Bar plots showing different cell–cell interaction types from Schwann cells to other cell types between groups. (G) Dot plots showing the expression levels of CCL family chemokines in different cell types in human HNSCC samples. (H) Violin plots showing the expression levels of *CCL2* in HLA‐DR^+^ and HLA‐DR^−^ Schwann cells in human HNSCC samples. (I) MRNA expression levels of Il1β. Mph and Trem2. Mph marker genes in THP‐1‐derived macrophages treated with or without rhCCL2 (100 ng/mL). (J‐K) Representative images of IF staining of Il1β (J) and quantitative results of Il1β fluorescence intensity (K) in THP‐1‐derived macrophages treated with rhCCL2 in different concentration (n = 5). Scale bar, 25 µm. Red: Il1β, blue: Dapi. (L) MRNA expression levels of Il1β. Mph and Trem2. Mph marker genes in THP‐1‐derived macrophages treated with conditional medium from HLA‐DR^−^ and HLA‐DR^+^ Schwann cells. (M‐N) Representative images of IF staining of Il1β (M) and quantitative results of Il1β fluorescent intensity (N) in THP‐1‐derived macrophages with different treatments (n = 5). Scale bar, 25 µm. Red: Il1β, blue: Dapi. *P* values were calculated by one‐side Wilcoxon test in H, by two‐sided Student's *t*‐test in (I,K,L,N), and by two‐sided log‐rank test in (C). ^*^ *p* < 0.05, ^**^ *p* < 0.01, ^***^ *p* < 0.001.

To investigate the mechanism by which HLA‐DR^+^ Schwann cells facilitate HNSCC progression, we performed scRNA‐seq on tumor samples from each group (MHC II‐NC and MHC II‐shRNA). ScRNA‐seq yielded 13, 109 cells after quality control and filtering (Figure 5A,B; Figure S8A). GO and KEGG analyses revealed that Schwann cells upregulate genes related to the neurotrophin signaling pathway and metabolism pathways in the MHC II‐shRNA group compared to those in the MHC II‐NC group, which indicated that the Schwann cells in MHC II‐shRNA group partially reversed the biological characteristics (Figure S8B,C). Furthermore, upon depleting HLA‐DR^+^ Schwann cells, the presence of tumor‐infiltrating Tregs was significantly inhibited (count: *p* < 0.0001, ratio: *p* = 0.0002), and the Treg score of CD4 Th cells also decreased (Figure S8D–F), which was consistent with previous in vitro results that HLA‐DR^+^ Schwann cells shaped the Treg‐related immunosuppressive TME.

Notably, we observed that Il1β. Mph was enriched in MHC II‐NC group, which was validated via mIF assay (Figure 5B; Figure S9A–C). GO and KEGG pathway analyses revealed that marker genes of Il1β. Mph were enriched in “NF‐κB signaling pathway”, “TNF signaling pathway”, “chemokine signaling pathway”, and “regulation of immune system process” (Figure S9A,B) and Il1β. Mph was associated with poorer OS in TCGA‐HNSC cohort (*p =* 0.034, Figure 5C). In the in‐house human TMA cohort, we validated the presence of Il1β. Mph in HLA‐DR^+^ Schwann cell‐rich samples and found that the Il1β. Mph infiltration ratio is positively correlated with the HLA‐DR^+^ Schwann cell ratio (R = 0.72, *p* < 0.0001, Figure 5D,E). Based on the close relevance of Il1β. Mph with HLA‐DR^+^ Schwann cells and their clinical outcome significance, we focused on this macrophage subtype and investigated the mechanism of how HLA‐DR^+^ Schwann cells generated them.

Via the CellChat algorithm, we analyzed the potential cell–cell interaction from Schwann cells to other cell types and found that CCL signaling pathway was the top interaction mode in HLA‐DR^+^ Schwann cells (Figure 5F). To validate results in the mouse data, we screened chemokine genes including CCL family and CXCL family in different cell types in the human scRNA‐seq data. Notably, although HLA‐DR^+^ Schwann cells seldom expressed other chemokines such as *CXCL9* or *CXCL10*, they exhibited a higher expression level of *CCL2* than HLA‐DR^−^ Schwann cells and other immune cells in the TME (Figure 5G,H; Figure S9D,E). Consistently, *ccl2* was found to be significantly inhibited in Schwann cells in the MHC‐shRNA group (Figure S9F). Subsequently, we conducted following in vitro experiments: recombinant human CCL2 (rhCCL2, 100 ng/mL) was added into the medium of THP‐1‐derived macrophage and we observed that rhCCL2 supplementation resulted in the upregulation of Il1β. Mph markers (*Il1β*, *CCL5*, *CXCL1*, *CXCL2*, *CXCL3*, *CXCL10*, and *TNFα*) and the downregulation of the Trem2 Mph marker (*Trem2*) in macrophages (Figure 5I). The results of Immunocytochemistry (ICC) assay showed an increase of Il1β expression in macrophages treated with rhCCL2 in a concentration‐dependent manner, which further indicated that CCL2 could induce the expression of Il1β (Figure 5J–K). In the meantime, the conditional media (CM) of HLA‐DR^−^ and HLA‐DR^+^ Schwann cells were also collected and used for macrophage incubation. Although the CM of cancer cells (CM‐Tumor) or HLA‐DR^−^ Schwann cells (CM‐HLADR^−^) failed to induce marker genes of Il1β. Mph, incubation with CM of HLA‐DR^+^ Schwann cells (CM‐HLADR^+^) led to an upregulation of these genes (Figure 5L; Figure S9G). Specifically, the administration of the CCL2 inhibitor, Pirfenidone, significantly alleviated the Il1β. Mph generation effect of CM‐HLADR^+^, which suggested that HLA‐DR^+^ Schwann cells induce Il1β. Mph transformation via CCL2 (Figure 5M,N).

### 2.6 HLA‐DR^+^ Schwann Cells Generate the Treg‐Related Niche with the Assistance of Il1β. Mph

**FIGURE 6:**
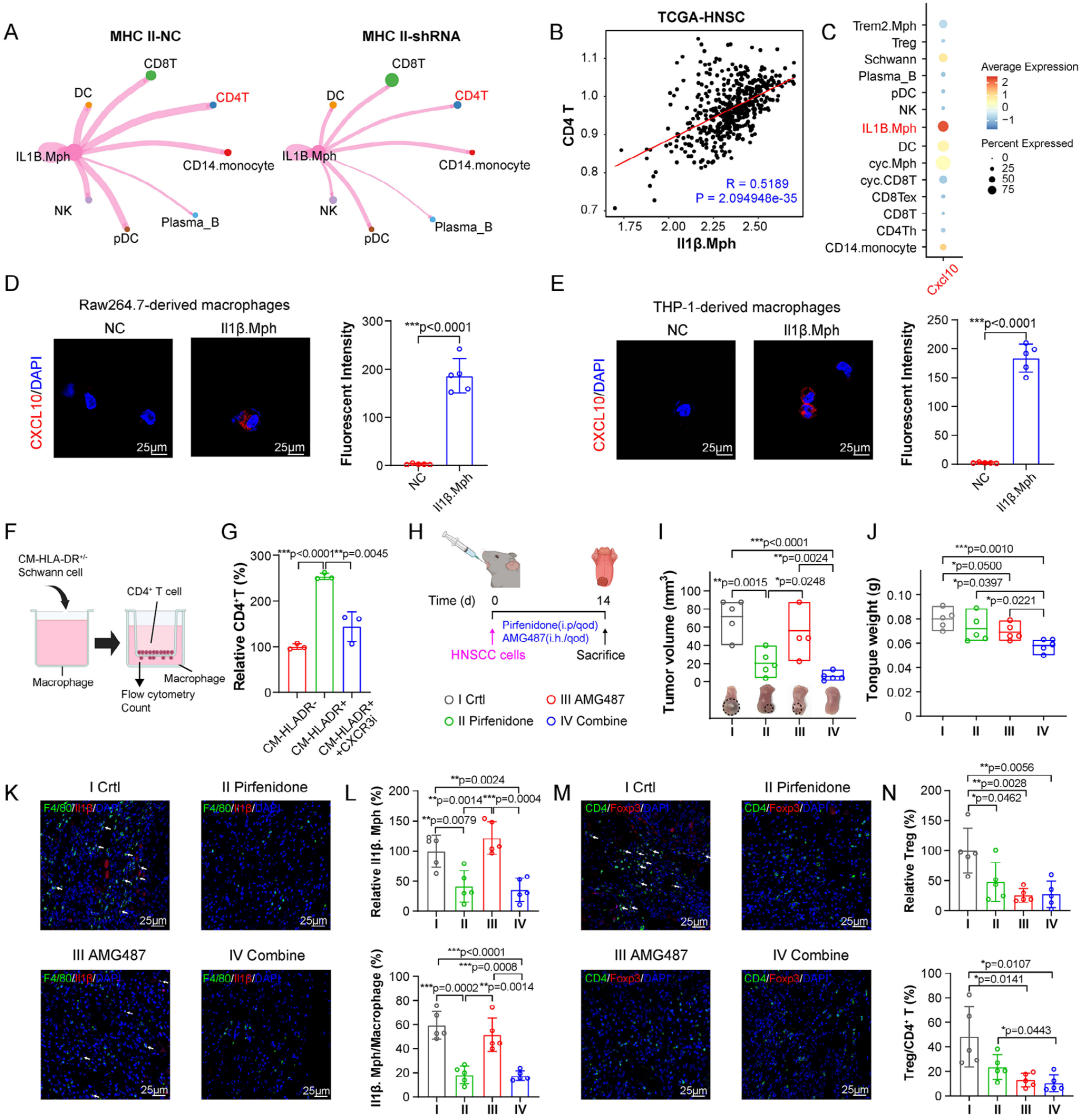
HLA‐DR^+^ Schwann cells generate the Treg‐related niche with the assistance of Il1β. Mph. (A) Circle plots showing the cell–cell interaction strength of Il1β. Mph and other immune cells in the MHC II‐NC and MHC II‐shRNA groups. (B) The Pearson correlation results of Il1β. Mph infiltration level and CD4^+^ T cell infiltration level in the TCGA‐HNSC cohort. (C) Bubble plots showing the expression levels of *Cxcl10* in different cell types. (D‐E) Representative images of IF staining of CXCL10 (left) and quantitative results of CXCL10 fluorescence intensity (right) in Raw264.7‐derived (D) and THP‐1‐derived (E) macrophages (n = 5). Scale bar, 25 µm. Red: CXCL10, blue: Dapi. (F‐G) Schematic images (F) and quantitative results (G) of CD4^+^ T cell migration assay (n = 3) (created with BioRender.com). (H) Schematic images of the orthotopic tumor xenograft model (created with BioRender.com). (I,J) Tumor volumes (I) and tumor weights (J) from mice in different groups (n = 5). I: Control, II: Pirfenidone, III: AMG487, IV: combination of Pirfenidone and AMG487. (K,L) Representative mIF images of Il1β. Mph (K) and quantitative results of Il1β. Mph counts (L, upper) and ratios (L, lower) in tumor samples from different groups (n = 5). Scale bar, 25 µm. Green: F4/80, red: Il1β, blue: Dapi. Il1β. Mphs are indicated by white arrows. (M‐N) Representative mIF images of Tregs (M) and quantitative results of Treg counts (N, upper) and ratio (N, lower) in tumor samples from different groups (n = 5). Scale bar, 25 µm. Green: CD4, red: Foxp3, blue: Dapi. Tregs are indicated by white arrows. *P* values were calculated by two‐sided Student's *t*‐test in (D,E,G,I,J,L,N). ^*^ *p* < 0.05, ^**^ *p* < 0.01, ^***^ *p* < 0.001.

We have identified strong correlations between HLA‐DR^+^ Schwann cells and Tregs and HLA‐DR^+^ Schwann cells and Il1β. Mph. Next, we were interested in the role of Il1β. Mph in the HLA‐DR^+^ Schwann cell niche. Via the CellChat algorithm, we observed a stronger interaction between Il1β. Mph and CD4^+^ T cells in MHC II‐NC mice compared to MHC II‐shRNA mice (Figure 6A), which was validated in the TCGA‐HNSC cohort (R = 0.5189, *p <* 0.0001, Figure 6B). As shown in Figure 5I,L, *CXCL10* was the most upregulated marker gene in the Il1β. Mph induction model and exhibited relatively specific expression in Il1β. Mph (Figure 6C). We validated the CXCL10 expression in both mouse (Raw264.7) and human (THP‐1) Il1β. Mph (Figure 6D,E). CXCL10 is considered as a classic chemokine molecule and could recruit T cell by interacting with CXCR3 [31]. Therefore, we hypothesized that Il1β. Mph may enhance CD4^+^ T cell infiltration via CXCL10/CXCR3. The hypothesis was subsequently validated by the coculture assay: macrophages were seeded in the wells and CM from HLA‐DR^−^ or HLA‐DR^+^ Schwann cells was added to the cell medium for Il1β. Mph induction. Afterwards, Transwell devices with CD4^+^ T cells was placed into the well. After 12 h of coculture, CD4^+^ T cells in the lower chamber were collected for flow cytometry analysis (Figure 6F). In the meantime, we also evaluated CD25^+^ and Foxp3^+^ CD4^+^ T cell ratio in the coculture assay as described previously to investigate whether Il1β. Mph could generate Treg cells from CD4^+^ T cells. We found Treg phenotypes were comparable between Il1β. Mph and control Mph groups but Il1β. Mph was able to recruit more CD4^+^ T cells (*p* < 0.0001) than control macrophages (Figure 6G; Figure S10A,B). Notably, when the CXCR3 inhibitor AMG487 was used to inhibit the combination of CXCL10 and CXCR3 [32], the numbers of CD4^+^ T cells that migrated to the Il1β. Mph significantly decreased (*p* = 0.0045), which suggested that Il1β. Mph recruited Tregs via CXCL10/CXCR3. Given that HLA‐DR^+^ Schwann cells hardly expressed chemokines except for *CCL2*, they are not able to recruit CD4^+^ T cells by themselves (Figure 5G; Figure S9D). However, with the assistance of Il1β. Mph, HLA‐DR^+^ Schwann cells were able to transform infiltrated CD4^+^ T cells into Tregs and thus generate an immunosuppressive niche.

Finally, we assessed the effect of tumor‐neuron‐immune niche on HNSCC progression in vivo in an orthotopic tumor xenograft model (Figure 6H). Mouse HNSCC cells (SCC7) were orthotopically injected into the mouse tongue, and the mice were then randomly divided into four groups: the control group (group I), which was treated with saline (i.p.) every 2 days; the Pirfenidone group (group II), which was treated with Pirfenidone group (i.p.) every 2 days (reported as a CCL2 inhibitor [33]); the AMG487 groups (group III), which was treated with AMG487 (i.h.) every 2 days (reported as a CXCL10/CXCR3 inhibitor); and the combination group (group IV), which was treated with Pirfenidone and AMG487. We found that Pirfenidone treatment significantly suppressed tumor sizes (II vs I, *p* = 0.0015, Figure 6I) and AMG487 treatment significantly decreased tumor weights (III vs I, *p* = 0.0397, Figure 6J). Although mono‐therapeutic did not achieve satisfactory tumor inhibition results in both tumor size and tumor weights, the combination of Pirfenidone and AMG487 resulted in superior tumor suppression results (IV vs I, volume: *p* < 0.0001, weight: *p* = 0.0010), which indicated that the interfering HLA‐DR^+^ Schwann cell‐Il1β. Mph or Il1β. Mph‐CD4^+^ T interactions can partially alleviate HNSCC growth while inhibiting HLA‐DR^+^ Schwann cell‐Il1β. Mph‐Treg interplay achieved significant antitumor effects. Moreover, Pirfenidone and AMG487 treatment also substantially altered the immune microenvironment of HNSCC. Specifically, fewer Il1β. Mph (II vs I, Figure 6K,L) and Tregs (II vs I, Figure 6M,N) were observed in the Pirfenidone group, which validated the presence of Il1β. Mph generation effects of CCL2 and the Treg transformation effects of HLA‐DR^+^ Schwann cells. Interestingly, AMG487 treatment did not affect Il1β. Mph infiltration in the TME (III vs I, Figure 6K,L), but decreased Treg infiltration (III vs I, Figure 6M,N), which suggested that Tregs play a downstream effector role in the Schwann cell‐Il1β. Mph‐CD4^+^ T interplay. Overall, we concluded that HLA‐DR^+^ Schwann cells generated Treg‐related niche with the assistance of Il1β. Mph and facilitate HNSCC progression.

## 3 Discussion

Recent advances in cancer neurology have revealed an oncogenic function of peripheral nerves in cancer initiation and progression [34, 35]. Notably, many studies have shown an intimate association between neural components and HNSCC: neural cells can promote HNSCC growth, influence the TME of HNSCC, and be used to construct a prediction model of HNSCC patient outcomes [13, 14, 16, 17, 18], which indicates the active and crucial role of neural cells in HNSCC and highlights the importance of investigating neural structures in understanding tumor activities. In this study, we analyzed scRNA‐seq data of samples from HNSCC patients, including normal tissues, precancerous tissues, and tumor tissues. We annotated Schwann cells in these samples, and for the first time, we constructed the dynamic transition trajectory of tumor‐associated Schwann cells along cancer initiation and progression: Schwann cells downregulated neural‐related signatures (e.g., Neuroactive ligand‐receptor interaction), but upregulated expression levels of immunoregulatory pathways (e.g., Antigen processing and presentation, immune checkpoint inhibition ligand, Treg). Among enriched function pathways in Schwann cells in HNSCC samples, antigen processing and presentation ranked as the top, with the expression levels of HLA‐DR genes consistently increased along the trajectory. Therefore, we further identified a Schwann cell subpopulation, HLA‐DR^+^ Schwann cells, which was validated in the in‐house validation cohort and found to be associated with poorer outcomes of HNSCC patients in both public and in‐house cohorts.

According to previous studies in other neural‐rich tumors, Schwann cells function as paths of axonal guidance toward tumor cells and contribute to neurogenesis in the TME [36]. In addition, Schwann cells increase the migratory, invasive, and metastatic abilities of tumor cells via oncogenic signaling pathways such as the PI3K/AKT and Snail‐Twist pathways [5, 37]. However, different from previous reports, in the current study, we found comprehensive TME remodeling effects of HLA‐DR^+^ Schwann cells in HNSCC, not only activating cancer cell activities but also influencing the whole immune microenvironment. First, as for the cancer‐neuron crosstalk, we used direct and indirect coculture assay, mIF assay, flow cytometry and found that HLA‐DR^+^ Schwann cells were hijacked and educated by HNSCC cells via NRG1/ERBB3 axis; In turn, HLA‐DR^+^ Schwann cells enhanced ECM‐receptor interaction pathway in HNSCC cells. Next, as for the neuron‐immune interplay, HLA‐DR^+^ Schwann cells shaped a Treg‐related immunosuppressive niche. To be more specific, they can drive CD4^+^ T cells into Tregs by MHC‐II molecules, which was validated via in vitro coculture assay, immunophenotyping, and in vivo gene engineering mice models. According to the canonical theory, MHC II molecules are expressed in pAPCs such as DCs and macrophages, which are able to completely active CD4^+^ T cells with the assistance of costimulatory signals [38]. However, we found that HLA‐DR^+^ Schwann cells rarely express these costimulatory signals compared to other immune cells in HNSCC, which suggested that they may function as amateur APCs (aAPCs) with an inefficient ability to activate T cells while inducing CD4^+^ T cells to transform into dysfunctional Tregs [39]. Interestingly, although HLA‐DR^+^ Schwann cells exhibited low expression levels of most chemokines, which suggested limited immune recruitment capability of them, they can induce Il1β. Mph via *CCL2*. Il1β. Mph is a subtype of macrophage, with high expression of *CXCL10* and enriched marker genes related to chemokine signaling pathway, and was identified as a protumor subpopulation in multiple cancers [40, 41]. Il1β. Mph promoted CD4^+^ T cell accumulation via *CXCL10/CXCR3* axis and fuel the generation of Tregs by them. In this way, a comprehensive cancer‐neuron‐immune niche came into formation and facilitated HNSCC progression.

Several limitations of this study should be acknowledged. First, as for the technical concern, the tissue processing operations may have inevitably bias against Schwann cells, which could lead to the insufficient dissociation and relative low cell capture of Schwann cells. Second, when constructing the clinical cohort, sex issue was not considered carefully and in the animal experiments, only male mice were used, which could lead to the sexual bias. Additional caution should be taken when extrapolating general conclusions to both sexes. Accumulating evidence has demonstrated significant gender disparities in immune response and disease susceptibility, which may affect the generalizability and extrapolation of our experimental findings. Therefore, we should pay more attention on gender ratio of clinical samples and integrate both male and female experimental animals to further verify our conclusions and eliminate the confounding influence of gender factors. Third, the survival analysis was performed using an optimal cutoff value to dichotomize the signature. Such binary categorization based on cutoff points may introduce potential bias and information loss. Moreover, we used small‐molecule inhibitors to block cancer‐neuron‐immune interplay in the mouse model, but these drugs were still at a proof‐of‐concept level and have broad biological effects. The nonspecific inhibition in other cells may impact the results.

## 4 Conclusions

The present study delineated the dynamic transition of Schwann cells during cancer initiation and progression and identified a Schwann cell subpopulation, HLA‐DR^+^ Schwann cell, that hijacked by cancer cells and enriched as HNSCC progression. This study revealed that HNSCC cells educate Schwann cells via NRG1/ERBB3 and activate the JAK‐STAT signaling pathway in Schwann cells to upregulate HLA‐DR expression. HLA‐DR^+^ Schwann cells induced a macrophage subpopulation, Il1β. Mph, which promoted CD4^+^ T cells accumulation for HLA‐DR^+^ Schwann cells and assisted them in shaping the cancer‐neuron‐immune niche and facilitating HNSCC progression. Our findings revealed the mechanism of how HLA‐DR^+^ Schwann cells reprogrammed the TME of HNSCC, provided insights into tumor neurology, and laid the foundations for therapeutics development targeting the cancer‐neuron‐immune niche of HNSCC (Figure S11).

## 5 Experimental Section

### 5.1 Ethical Statement

This study was reviewed and approved by the local medical ethics committee of Shanghai Ninth People's Hospital Affiliated with Shanghai Jiao Tong University. Written informed consent was obtained from each patient prior to sample collection.

### 5.2 Clinical Cohort Information

The detailed demographic and clinical information of the in‐house scRNA‐seq cohort and in‐house validation cohort are described in the previous study [19].

### 5.3 Immunohistochemistry (IHC) Staining

Formalin‐fixed, paraffin‐embedded (FFPE) tumor and normal tissues were prepared for tissue microarray as previously reported [19]. The slides were baked at 65°C overnight. After deparaffinization and hydration, these slides were boiled in citrate buffer at 100°C for 15 min. Subsequently, a 3% H_2_O_2_ solution was used to block endogenous peroxidase activity for 20 min. To prevent nonspecific antibody binding, the slides were then incubated with 5% normal goat serum for 1 h at room temperature. Then these slides were incubated with primary antibodies at 4°C overnight. Following 3 washes with TBST, the slides were incubated with an HRP‐conjugated goat anti‐rabbit/mouse secondary antibody (GeneTech, #GK500705) for 1 h at room temperature. The sections were stained with DAB (Vector, #PK‐6100) and then counterstained with hematoxylin according to the manufacturer's instructions. The 3Dhistech Pannoramic Scan system was used for image acquisition. A pathologist performed semiquantitative analysis of IHC staining in a blinded manner by calculating the percentage of positive cells. Primary antibodies used in the current investigation are listed below: anti‐Foxp3 (Abcam, #Ab20034, 1:500), anti‐CD4 (Abcam, #Ab133616, 1: 250), anti‐CD8 (Abcam, #Ab101500, 1:100), anti‐CD68 (Abcam, #Ab955, 1:3000), anti‐CD11c (Abcam, #Ab52632, 1:200), and anti‐CD20 (Abcam, #Ab78237, 1:100).

### 5.4 Multiplex Immunofluorescence (mIF) Staining

mIF staining of 4‐µm FFPE sections was performed using the PANO 4‐plex IHC kit (Absin, #Abs50012) according to the manufacturer's instructions. Different primary antibodies were sequentially applied, followed by HRP‐conjugated secondary antibody incubation and tyramide signal amplification. The slides were microwave heat‐treated following each round of tyramide signal amplification. Nuclei were stained with DAPI (Sigma, #D9542) after labeling antigens. The following antibodies were used for human samples: anti‐S100B (Abcam, #Ab52642, 1:100), anti‐HLA‐DR (Abcam, #Ab92511, 1:100), anti‐CK5 (Abcam, #Ab52635, 1:100), anti‐NRG1 (Proteintech, #10527‐1‐AP, 1:200), anti‐CD4 (Abcam, #Ab133616, 1:400), anti‐CD68 (Abcam, #Ab955, 1:1000), and anti‐Il1β (Proteintech, #16806‐1‐AP, 1:500). The following antibodies were used for mouse samples: anti‐S100B (Abcam, #Ab52642, 1:100), anti‐IA/IE (Biolegend, #107625, 1:100), anti‐CK5 (Abcam, #Ab52635, 1:100), anti‐TNC (Abcam, #Ab108930, 1:500), anti‐CD4 (Abcam, #Ab183685, 1:200), anti‐CD11c (Abcam, #Ab219799, 1:100), anti‐CD68 (Abcam, #Ab186525, 1:500), anti‐αSMA (Abcam, #Ab7817, 1:1000), anti‐Foxp3(Abcam, #Ab215206, 1:100), anti‐F4/80 (Abcam, #Ab300421, 1:5000), and anti‐Il1β (Abcam, #Ab283818, 1:500). The 3Dhistech Pannoramic Scan system was used for image acquisition.

### 5.5 Single‐Cell RNA Sequencing (scRNA‐seq)

scRNA‐seq of human samples has been described in our previous study [19]. As for the mouse samples, tongue tumors were harvested from three mice in the indicated group, and then processed to isolate single cells using collagenase digestion. These isolated cells were subsequently subjected to scRNA‐seq analysis utilizing the 10x Genomics platform and sequenced on the Illumina NovaSeq 6000, with an aim to obtain 50 000 reads per cell. The raw data underwent quality control, mapping, and count table assembly using the CellRanger pipeline version 7.0.

### 5.6 Data Preprocessing

scRNA‐seq data preprocessing was performed by NovelBio Co., Ltd. with NovelBrain Cloud Analysis Platform (www.novelbrain.com). We applied fastq [42] with default parameter filtering of the adaptor sequence and removed low‐quality reads to obtain clean data. Then, feature‐barcode matrices were obtained by aligning reads to the mouse genome mm10 using CellRanger (v3.1.0). We performed a downsample analysis of the samples sequenced according to the mapped barcoded reads per cell of each sample, and finally achieved the aggregated matrix. Cells containing more than 200 expressed genes and a mitochondrial UMI percentage less than 10% passed cell quality filtering, and mitochondrial genes were removed from the expression table.

### 5.7 Dimension Reduction and Clustering Analysis

Dimension reduction and unsupervised clustering were performed according to the standard workflow in Seurat (v4.13) [43]. To integrate cells from different samples into a shared space for unsupervised clustering, we used the IntegrateDatafunction algorithm in the Seurat R package (v4.13) to perform batch effect correction. For clustering and visualization, we applied the *FindCluster* function in *Seurat* to obtain cell clusters at various resolutions and reduced the dimensionality of the data using Uniform manifold approximation and projection (UMAP) implemented in the *RunUMAP* function with the following settings: reduction = ‘pca’, dims = 1:20.

### 5.8 Analysis of Differentially Expressed Genes (DEGs)

We applied the *FindMarkers* function in Seurat to identify DEGs between two groups with the min.pct parameter set at 0.1, which considers only genes expressed in more than 10% of cells. The method MAST was used to obtain the *p* value for comparisons, which uses a Gaussian hurdle model to combine both differences in detection rate and differences in mean, and the adjusted *p* value based on Bonferroni correction was calculated. Genes with adjusted *p* < 0.05 and absolute log_2_|fold change| > 0.2 were considered differentially expressed.

### 5.9 Functional Annotation Analyses

Kyoto Encyclopedia of Genes and Genomes (KEGG) and Gene Ontology (GO) enrichment analyses were carried out for DEGs between two groups or target genes of cell‐to‐cell communication by the R package *clusterProfiler* (v4.0.5) [44]. We considered pathways with *p* < 0.05 to be significantly enriched. To characterize the relative activation of a given gene set, such as neural function and immunoregulatory pathways, we performed *AddModule* function of Seurat.

### 5.10 Single‐Cell Trajectory Reconstruction and Analysis

To characterize the potential phenotypic changes of Schwann cells, we performed trajectory analysis of with monocle2 (v2.24.0; parameters: method = “DDRTREE”, orderinggenes = marker genes [45, 46, 47]). Then, the cell differentiation trajectory was inferred after dimensionality reduction and cell ordering. Branched expression analysis modeling was used to further test for branch‐dependent gene expression. Genes that changed along the pseudotime axis were calculated and are visualized as a branched heatmap. Genes with similar expression were clustered, and the group of genes was functionally annotated.

### 5.11 Cell–Cell Interaction Analysis

CellChat (v1.6.1) was used to infer cell–cell communication by integrating scRNA‐seq data with the ligand‐receptor interaction database CellChatDBHuman [48]. CellChat quantifies the communication probability between two interacting cell groups based on the average expression values of a ligand and a receptor as the cofactors. The calculated communication probabilities are assigned as edge weights to quantify the interaction strength. Additionally, CellChat computes the communication probability at the signaling pathway level by summarizing the communication probabilities of all ligand‐receptor interactions associated with each signaling pathway. To identify signaling changes, the differential outgoing and incoming interaction strengths of this cell population in each cell–cell communication network between the two conditions were calculated and compared. The R package NicheNetR (v2.0.6) [49] was also used to infer mechanisms of interaction. For ligand and receptor interactions, clustered cells with gene expression over 10% were considered. The top 100 ligands and top 1000 targets of DEGs of “sender cells” and “receiver cells” were extracted for paired ligand‐receptor activity analysis. The function ligand_activity_target_heatmap in Nichenet_output was used to display the regulatory activity of ligands.

### 5.12 Survival Analysis in the TCGA‐HNSC Cohort

The “*surv_cutpoint*” function of R package survminer, an outcome‐oriented method providing a value of a cut‐point that corresponds to the most significant relation with outcomes, was used to perform dichotomy of indicated signature expression and to divide the patients into two groups according to the selected maximum logarithm statistics. The gene signatures used for survival analysis can be found in Table S1. The two‐sided long‐rank test was used to compare Kaplan–Meier survival curves. The hazard ratio (HR) was calculated by the Cox proportional hazards model by R package survival (v3.3.1), and the 95% CI is reported. Multivariable Cox regression was performed by R package survival (v3.3.1) by considering the confounding factors.

### 5.13 Estimation of Correlations of Different Signatures

We calculated correlations of different signatures in TCGA‐HNSC cohort by Pearson's correlation test. The tumor biological activity signatures, HLA‐DR^+^ Schwann cell signature, Il1β.Mph signature, CD4^+^ T signature, and Treg signature expression can be found in Table S1.

### 5.14 Cell Lines

Human Schwann cell sNF96.2 and mouse Schwann cell SW10 were obtained from American Type Culture Collection (Manassas, VA, USA). Human HNSCC cell line Cal27, mouse HNSCC cell line SCC7, and human monocyte cell line THP‐1 were obtained from FuHeng Biology Company (Shanghai, China) as described previously [50]. Cell lines were tested negative for mycoplasma contamination. No misidentified lines were used. Human CD4^+^ T cells were isolated from peripheral blood of healthy donors using the EasySep Human CD4^+^ T‐Cell Isolation Kit (Stemcell, #17952). Mouse CD4^+^ T cells were isolated using a CD4^+^ T cell isolation kit (Stemcell, #19852) from splenocytes of the 8‐week‐old male Balb/c mice.

SNF96.2, SW10, and Cal27 cells were cultured in DMEM medium (GIBCO, #C11995500BT) supplemented with 10% fetal bovine serum (FBS) and 1% penicillin‐streptomycin. SCC7, THP‐1, and T cells were cultured in Roswell Park Memorial Institute (RPMI) 1640 medium (GIBCO, #C11875500BT) supplemented with 10% FBS and 1% penicillin‐streptomycin. ImmunoCult Human CD3/CD28 T Cell Activator (Stemcell, #10971) and Dynabeads Mouse T‐Activator CD3/CD28 (Thermofisher, #11453D) were added into T cell medium (25 µL/mL) for human and mouse T cell activation, respectively, and human recombinant IL‐2 (Stemcell, #78036) was added into T cell medium (50 IU/mL) for cell expansion. To induce macrophage differentiation, THP‐1 cells were seeded at a density of 10^6^ cells in a six well plate in the presence of 20 ng/ml PMA (MCE, #HY‐18739) for 18 h, rested in fresh THP‐1 medium for 8 h and were then incubated in fresh THP‐1 medium for 72 h [51].

### 5.15 Transfection

SiRNAs specific for *NRG1* and *CIITA* were purchased from Shanghai Genepharma Co., Ltd (Shanghai, China). A scrambled nontargeting siRNA was used as negative control. Plasmid specific for *CD80* and *CD86* were purchased from Ibsbio Co., Ltd. (Shanghai, China). The cells were transfected with 100 nm siRNAs diluted in Opti‐MEM (Thermo Fisher, #31985070) using Lipofectamine 3000 (Thermo Fisher, #L3000015) according to manufacturer's protocols. For plasmid transfection, lipofectamine 3000 and plasmid with a proportion of 2.5:1 was diluted with 250 µL serum‐free Opti‐MEM respectively.

### 5.16 Coculture of Tumor Cells, Schwann Cells, Macrophages, and CD4^+^ T Cells In Vitro

For direct coculture of tumor cells and Schwann cells, 2 × 10^5^ HNSCC cells and 2 × 10^5^ Schwann cells were mixed and seeded in six‐well plates overnight. 2 × 10^5^ Schwann cells were set as control. For the indirect coculture of tumor cells and Schwann cells based on Transwell devices, 2 × 10^5^ Schwann cells were seeded in the lower compartment of six‐well plates and 2 × 10^5^ HNSCC cells were seeded on top of the Transwell membrane (Corning, 0.4 µm). As for the *NRG1* knockdown group, HNSCC cells were transfected with corresponding siRNA first (siNC as control) for 24 h and then seeded on the Transwell devices for coculure; as for the αERBB3 and the JAK/STAT signaling pathway inhibitor group, αERBB3 (Selleck, #A2506, 5 mg/mL) and WP1066 (MCE, #HY‐15312, 5 µm) were directly added into the culture medium of Schwann cells respectively. After cocultured for 24 h, Schwann cells were collected for further analysis.

For coculture of Schwann cells and total CD4^+^ T cells, 2 × 10^5^ Schwann cells were seeded in six‐well plates and were transfected with corresponding siRNAs or plasmids (siNC or vector plasmid as control). After 24 h, Schwann cells were cocultured with tumor cells first with Transwell devices as described above. Then, 2 × 10^5^ total CD4^+^ T cells were added into the plates and cocultured with Schwann cells for 48 h. After coculture, CD4^+^ T cells were collected for further analysis.

For coculture of tumor cell‐derived conditional medium (CM) and macrophages. HNSCC cells were cultured in standard medium in the six‐well plates until reaching 80%–90% confluence. Thereafter, the medium was replaced with 2 mL DMEM without FBS or phenol red and cultured for another 24 h. Then, the cell culture medium was harvested and was used to incubate THP‐1‐derived macrophages. Pirfenidone (MCE, #HY‐B0673, 0.5 mg/mL) was added to HNSCC culture medium to inhibit CCL2 secretion.

For the coculture of Il1β. Mph and CD4^+^ T cells, 2 × 10^5^ macrophages were pre‐seeded in the lower chamber and were treated with CM from HLA‐DR^−^ or HLA‐DR^+^ Schwann cells to induce Il1β. Mph (CM from HLA‐DR^−^ Schwann cells as control). 1 × 10^6^ CD4^+^ T cells were seeded on the top chamber of the 24‐well plate Transwell system (Corning, 3 µm). AMG487 (MCE, #HY‐15319, 100 nm) was added to macrophage culture medium to inhibit CXCL10/CXCR3 combination. 24 h later, cells from the top chamber and migrated toward the tumor cells were collected for flow cytometry to enumerate transmigrated cell counts in different groups.

### 5.17 Immunocytochemistry (ICC) Analysis with Confocal Microscopy

Macrophages were seeded into confocal dishes (NEST, #801001) with a φ of 20 mm. Then, recombinant human CCL2 (rhCCL2) (MCE, #HY‐P700034AF) at different concentrations or CM from different groups were added into the cell medium. After 24 h, cells were fixed with 4% paraformaldehyde for 15 min, permeabilized in 0.1% Triton X‐100 or saponin for 10 min, and then blocked with 1% BSA for 30 min. Primary antibody anti‐Il1β (Abcam, #Ab283818, 1:50) or anti‐CXCL10 (ThermoFisher, #701225, 1:200) was added and incubated overnight at 4°C. After washing three times, the cells were then treated with Alexa Fluor 594‐conjugated secondary antibodies (Jackson, #111‐585‐003) for 1 h at room temperature and subjected to nuclear staining with DAPI (Sigma, #D9542) for 10 min. Finally, images were captured with a Confocal laser scanning microscope (CLSM, Leica SP8).

### 5.18 Flow Cytometry Analysis

As for Schwann analysis, Schwann cells were collected, incubated with indicated antibodies and analyzed by flow cytometry (BD Biosciences) according to the manufacture instructions. Antibodies used for human cells are listed below: anti‐S100B (Abcam, #Ab115803, 1:100), Goat Anti‐Rabbit IgG H&L (Abcam, #Ab150077, 1:2000), and anti‐HLA‐DR (Biolegend, #327014, 1:100). Antibodies used for mouse cells are listed below: anti‐S100B (Abcam, #Ab115803, 1:100), Goat Anti‐Rabbit IgG H&L (Abcam, #Ab150077, 1:2000), and anti‐IA/IE (Biolegend, #107625, 1:100).

As for CD4^+^ T cell immunophenotyping, CD4^+^ T cells were incubated with the indicated antibodies and analyzed by flow cytometry (BD Biosciences) according to the manufacture instructions. Antibodies are listed below: anti‐CD4 (Biolegend, #317408, 1:100), anti‐CD25 (Biolegend, #302612, 1:100), and anti‐Foxp3 (Biolegend, #320114, 1:100).

### 5.19 Western Blotting

The cell proteins in different groups were extracted from cultured cells using cold RIPA lysis buffer (Thermo Fisher, #89901) and protease inhibitor cocktail (Bimake, #B14001) and a phosphatase inhibitor cocktail (Bimake, #B15001). Proteins from cell lysates were separated by SDS‐PAGE and then transferred to PVDF membranes (Bio‐Rad, #1620175). The blots were incubated with primary antibodies at 4°C overnight and with secondary antibodies at room temperature for 1 h. Blot images were captured by ODYSSEY imaging system. Primary antibodies used in the current investigation were listed below: anti‐β‐actin (CST, #3700, 1:1000), anti‐STAT3 (CST, #9139, 1:1000), anti‐pSTAT3(CST, #9145, 1:2000), anti‐STAT2 (CST, #72604, 1:1000), anti‐pSTAT2 (CST, #88410, 1:1000), anti‐STAT1 (CST, #9172, 1:1000), anti‐pSTAT1 (CST, #7649, 1:1000), and anti‐JAK2 (CST, #3230, 1:1000).

### 5.20 RNA Isolation and Real‐Time qPCR Analysis

RNA was extracted from cell lines by using RNA isolation kit according to the manufacturer's instructions (Yeasen, #19221ES). Reverse transcription was performed using the PrimeScriptTM RT Reagent Kit with gDNA Eraser (TaKaRa, #RR047Q). Real‐time qPCR was conducted using SYBR Green qPCR Master Mix (Bimake, #B21702) and a QuantStudio 7 Flex Real‐ Time PCR System (Thermo Fisher). The primer sequences used in this study are listed in Table S2.

### 5.21 RNA Sequencing Analysis

In order to exclude tumor cell contamination, we selected indirect coculture assay for RNA sequencing of Schwann cells. As described before, after 24 h coculture, human and mouse Schwann cells were collected. Whole RNA was extracted from the indicated cell samples with a RNeasy Plus Mini Kit (Qiagen) according to the manufacturer's protocol. RNA was subjected to RNA‐Seq analysis on a BGISEQ‐500 system by Beijing Genomics Institute (BGI), China. In addition, the RNA was sheared and reverse transcribed through random primers to obtain cDNA for library construction. Subsequently, sequencing was performed on the prepared library [52]. All the generated raw sequencing reads were filtered to obtain clean reads stored in FASTQ format [53]. Bowtie2 and HISAT were used to map clean reads to reference genes and genomes, respectively [54]. RSEM was used to quantify the gene expression level (FPKM) [55]. The NOISeq method was used to screen out differentially expressed genes between two groups with a fold change ≥ 2 and divergence probability ≥ 0.8. GO and pathway annotation and enrichment analyses were based on the Gene Ontology Database (http://www.geneontology.org/) and KEGG pathway database (http://www.genome.jp/kegg/), respectively. The GO and KEGG pathway enriched analysis results could be seen in Tables S3 and S4.

### 5.22 Mice

All animal experiments were approved by the Animal Care and Use Committee of Ninth People's Hospital, Shanghai Jiao Tong University School of Medicine, Shanghai, China. 4‐week‐old C3H/HeJ mice were purchased from Beijing Vital River Laboratory Animal Technology Co., Ltd. (Beijing, China), and 4‐week‐old Balb/c nude mice were purchased from Shanghai Jihui Laboratory Animal Care Co., Ltd. (Shanghai, China). Only male mice were used in this study. All mice were maintained under pathogen‐free conditions in the animal care facilities of the Ninth People's Hospital, Shanghai Jiao Tong University School of Medicine. All animals were maintained at room temperature and with free access to food and water with a 12‐h light/dark cycle. All mice used in experiments throughout the study exhibited normal health. All mice were used after 1 week of acclimatization to the facility. All animal studies were performed in accordance with the Guide for Care and Use of Laboratory Animals (The Ministry of Science and Technology of China, 2006).

### 5.23 In Vivo Animal Studies

Subcutaneous tumor/Schwann cell co‐injection model was established using 4–6‐week‐old male Balb/c nude mice or C3H/HeJ mice. A total of 1 × 10^6^ SCC7 cells with or without 0.5 × 10^5^ Schwann cells in 100 µL PBS were inoculated subcutaneously into right flanks of mice as indicated [10]. The length (L) and width (W) of the tumors were measured every 2 days after injection, and the volume was calculated as (L × W^2^)/2. At the end point of the experiment, mice were sacrificed, and tumor tissues were collected for further analysis.

To generate Schwann cell‐targeted MHC II gene knockdown, 4‐week‐old male C3H/HeJ mice were randomly divided into two groups for administration of 50 µL of AAV (MHC II‐shRNA or MHC II‐NC) virus via orthotopically injection [9]. After 2 weeks, tumor cells were orthotopically transplanted into the tongue as previously reported [14]. Here, we prepared two batches of mice, one for long‐term survival observation and the other for short‐term tumor growth observation. Specifically, all mice underwent general anesthesia and were kept on a heating pad. A total of 1 × 10^5^ (long‐term observation) or 1 × 10^6^ (short‐term observation) SCC7 cells in 25 µL PBS were injected into the left side of the tongues of C3H/HeJ mice, and were monitored every 2 days. As for long‐term observation, mice were euthanized when body weight reduced by 20%; as for short‐term observation, mice were euthanized after 2 weeks of tumor cell injection, and tissue samples were collected for further analysis.

In the orthotopic tumor transplantation and pharmacological inhibition model, mice were orthotopically injected with 1 × 10^6^ SCC7 cells as described above. Then, they were randomly divided into four groups. Mice in the Pirfenidone group were intraperitoneally injected with 200 mg/kg every 2 days, mice in the AMG487 group were hypodermically injected with 5 mg/kg AMG487 every 2 days, mice in the combination group were administrated with Pirfenidone and AMG487 simultaneously, and mice in the control group were administrated with saline as negative control. Mice were euthanized after 2 weeks of tumor cell injection, and tissue samples were collected for further analysis.

### 5.24 Statistical Analysis

All data were shown as mean ± SD. The details about particular statistic parameters were specified in the figure legends and in the method section. All statistical analysis was performed using GraphPad Prism 8 software. *P* < 0.05 was considered as statistical significance, and the exact *p* values were indicated in the graphical data. Unless otherwise specified, the samples analyzed were independent biological replicates, and the two‐sided tests were used.

## Author Contributions

**Xiaoyan Meng** and **Zhonglong Liu** developed the study design, **Shijian Zhang** helped in preparing revised manuscript, **Xiaoyan Meng** and **Jingjing Sun** conducted experiments, **Luoman Gan** and **Liren Cao** assisted in experiments, **Lingfang Zhang** performed data analyses, and **Yue He** supervised the study. All the authors contributed to writing the manuscript.

## Conflicts of Interest

The authors declare no conflicts of interest.

## Supporting information




**Supporting File 1**: advs76131‐sup‐0001‐SuppMat.docx.

**Supporting File 2**: advs76131‐sup‐0002‐Table.xlsx.

## Data Availability

The data that supports the findings of this study are available in the supplementary material of this article.
