# Supplementary material for: HLA‐DR+ Schwann Cells Generate the Protumor Cancer‐Neuron‐Immune Niche in Head and Neck Squamous Cell Carcinoma
Source: Adv Sci (Weinh). 2026 Jun 15;13(51):e76131. doi: 10.1002/advs.76131 (PMC13336457; doi:10.1002/advs.76131)
Supplement: Supplementary file 1 [file ADVS-13-e76131-s002.docx]

Supporting Information

**HLA-DR^+^ Schwann cells generate the protumor cancer-neuron-immune niche in head and neck squamous cell carcinoma**

Xiaoyan Meng, Zhonglong Liu, Shijian Zhang, Luoman Gan, Liren Cao, Jingjing Sun, Lingfang Zhang, Yue He^*^

Dr. X. Y. Meng, Dr. Z. L. Liu, Dr. S. J. Zhang, L. M. Gan, L. R. Cao, Prof. Y. He

Department of Oral Maxillofacial & Head and Neck Oncology, Shanghai Ninth People’s Hospital, Shanghai Jiao Tong University School of Medicine; College of Stomatology, Shanghai Jiao Tong University; National Center for Stomatology; National Clinical Research Center for Oral Diseases; Shanghai Key Laboratory of Stomatology, Shanghai, P. R. China;

E-mail: william5218@126.com

Dr. S. J. Zhang

Department of Oral and Maxillofacial Surgery, Zhang Zhiyuan Academician Workstation, Hainan Province Clinical Medical Center for Stomatology, Hainan Western Central Hospital, Shanghai Ninth People’s Hospital, Danzhou, Hainan, P. R. China;

Department of Oral and Maxillofacial Head and Neck Oncology, Fengcheng Hospital of Shanghai Ninth People′s Hospital Group, Fengxian District, Shanghai, P. R. China;

Dr. J. J. Sun

Department of Oral Pathology, Shanghai Ninth People’s Hospital, Shanghai Jiao Tong University School of Medicine; College of Stomatology, Shanghai Jiao Tong University; National Center for Stomatology; National Clinical Research Center for Oral Diseases; Shanghai Key Laboratory of Stomatology, Shanghai, P. R. China;

L. F. Zhang

Suzhou Lingdian Biotechnology Co., Ltd, Suzhou, P. R. China;

**Figure S1** (A) Feature plots showing the Schwann cell cluster in GSE164241 (left) and GSE188737 (right), as indicated by arrows. (B) Box plots showing Schwann cell ratios in stromal cells in different stages. (C) Heatmap showing normalized expression levels of the indicated pathways in Schwann cells in different stages. (D) Expression levels of different scores of Schwann cells along the pseudotime axis. (E) Dot plots showing expression levels of HLA genes of Schwann cells in different stages. (F) Expression levels of HLA genes of Schwann cells along the pseudotime axis. (G) Dot plots showing markers of HLA-DR^+^ and HLA-DR^-^ Schwann cells. (H) Violin plots showing the expression level of *HLA-DRA* in myelinating and nonmyelinating Schwann cells. (I) The multivariable cox regression analysis of the TCGA-HNSC cohort. *P* values were calculated by two-sided Student’s *t*-test in B, H, and by two-sided log-rank test in I. ****p* < 0.001.

**Figure S2** (A) Bar plots showing the percentage of different clinical stages in patients with high (n = 26) and low (n = 26) HLA-DR^+^ Schwann cell infiltration in the validation cohort (n = 52). (B) The Kaplan-Meier MFS curves of samples with high (n = 26) and low (n = 26) HLA-DR^+^ Schwann cell infiltration in the validation cohort (n = 52). (C-D) Representative images of mIF staining of HLA-DR^+^ Schwann cell in mouse HNSCC tumor and normal tissue samples (C). Scale bar, 200 μm, 50 μm. Green: S100B, red: IA/IE, blue: Dapi. The quantitative results are shown in (D) (n = 80). (E-G) Representative images of mIF staining of Schwann cells in human intestinal (E), lung (F), and kidney (G) samples. Scale bar, 50 μm. Green: S100B, red: HLA-DR, blue: Dapi. *P* values were calculated by Chi square test in A, by two-sided Student’s *t*-test in D, and by two-sided log-rank test in B. **p* < 0.05, ***p* < 0.01, ****p* < 0.001.

**Figure S3** (A) Representative flow cytometry images of HLA-DR^+^ Schwann cell gating. (B) Relative expression levels of HLA-DR^+^ Schwann cell marker genes in Schwann cells in different groups (n = 3). (C-D) Representative flow cytometry images (left) and quantitative results of IA/IE^+^ cell ratio (right) of mouse Schwann cells directly cocultured (C) and indirectly cocultured (D) with HNSCC cells (n = 3). *P* values were calculated by two-sided Student’s *t*-test in B-D. **p* < 0.05, ** *p* < 0.01, ****p* < 0.001.

**Figure S4** Violin plots showing the *NRG1* (A) and *ERBB3* (B) expression level in different cell types in the scRNA-seq data.

**Figure S5** (A-B) GO (A) and KEGG pathway (B) analysis results of DEGs of human HLA-DR^+^ compared to HLA-DR^-^ Schwann cells. (C-D) GO (C) and KEGG pathway (D) analysis results of DEGs of mouse HLA-DR^+^ compared to HLA-DR^-^ Schwann cells. (E-F) Representative flow cytometry images (E) and quantitative results (F) of the HLA-DR^+^ cell ratio of Schwann cells subjected to different treatments (n = 3). *P* values were calculated by hypergeometric test in A-D, and by two-sided Student’s *t*-test in F. ****p* < 0.001.

**Figure S6** (A) Cell viability of mouse (left) and human (right) cancer cells cultured with or without Schwann cells (n = 5). (B) Tumor volumes (left) and tumor weights (right) of Balb/c nude mice injected with mouse HNSCC tumor cells with or without Schwann cells (n = 5). (C) Representative mIF staining of major organs of the MHC II-NC (upper) and MHC II-shRNA (lower) group. Scale bar, 1000 μm. Green: GFP, blue: Dapi. (D-E) Representative H&E staining of major organs (D) and tongues (E) from the MHC II-NC and the MHC II-shRNA groups. Scale bar, 50 μm, 1000 μm. (F-H) Representative images of mIF staining of I-A/I-E^+^ DCs (F), macrophages (G), and fibroblasts (H) in tumors from the MHC II-NC and MHC II-shRNA groups. Scale bar, 50 μm. Green: CD11c, CD68, αSMA, red: I-A/I-E, blue: Dapi. *P* values were calculated by two-sided Student’s *t*-test in A-B.

**Figure S7** (A-B) Representative images of mIF staining of HLA-DR^+^ Schwann cells (A) and IHC staining of different immune cells (B) in the validation cohort. Scale bar, 200 μm. Green: S100B, red: HLA-DR, blue: Dapi. (C) Pearson correlation results of HLA-DR^+^ Schwann cell count and different immune cell ratios in the validation cohort (n = 61). (D) The Pearson correlation results of Schwann cell infiltration level and Treg infiltration level in the TCGA-HNSC cohort. (E) Violin plots showing immune suppression scores in Schwann cell-high and -low samples in the TCGA-HNSC cohort. *P* values were calculated by two-sided Student’s *t*-test in E.

**Figure S8** (A) Dot plots showing the average expression of known markers in indicated cell clusters. (B-C) GO (B) and KEGG pathway (C) analysis of DEGs of Schwann cells of MHC II-shRNA compared to MHC II-NC group. (D) Violin plots showing Treg scores in CD4 Th cells in the MHC II-NC and the MHC II-shRNA groups. (E) Representative images of mIF staining of Treg cells in tumors from the MHC II-NC and MHC II-shRNA groups. Scale bar, 50 μm. Green: S100B, red: CD4, grey: Foxp3. (F) Quantitative results of Treg cell counts (upper) and ratio (lower) in tumors from the MHC II-NC and MHC II-shRNA groups (n = 20). *P* values were calculated by two-sided Student’s *t*-test in D, F, and by hypergeometric test in B, C. ***p* < 0.01, ****p* < 0.001.

**Figure S9** (A-B) GO (A) and KEGG pathway (B) analysis of marker genes of Il1β. Mph. (C) Representative images of mIF staining of Il1β. Mph in tumors from the MHC II-NC and MHC II-shRNA groups. Scale bar, 50 μm. Green: F4/80, red: Il1β, blue: Dapi. Quantitative results of Il1β. Mph ratio in tumors from the MHC II-NC and MHC II-shRNA groups were showed in the right (n = 20). (D) Dot plots showing the expression levels of CXCL family chemokines in different cell types in human HNSCC samples. (E) Violin plots showing the expression levels of *CCL2* in different cell types in human HNSCC samples. (F) Violin plots showing the expression levels of *CCL2* in Schwann cells in the MHC II-NC and the MHC II-shRNA groups. (G) MRNA expression levels of Il1β. Mph and Trem2. Mph marker genes in THP-1-derived macrophages treated with conditional medium from HNSCC cells. *P* values were calculated by two-sided Student’s *t*-test in C, by one-sided Student’s *t*-test in F, and by hypergeometric test in A-B. ****p* < 0.001.

**Figure S10** (A) Representative flow cytometry images of CD25^+^ CD4^+^ T cells (left) and Foxp3^+^ CD4^+^ T cells (right) cocultured with different macrophages (n = 3). (B) Quantitative results of CD25^+^ (left) and Foxp3^+^ (right) ratio. *P* values were calculated by two-sided Student’s *t*-test in B.

**Figure S11** Schematic illustration of the workflow and the mechanism of HLA-DR^+^ Schwann generating the tumor-promoting cancer-neuron-immune niche in HNSCC.
